# Mechanical loading of desmosomes depends on the magnitude and orientation of external stress

**DOI:** 10.1038/s41467-018-07523-0

**Published:** 2018-12-11

**Authors:** Andrew J. Price, Anna-Lena Cost, Hanna Ungewiß, Jens Waschke, Alexander R. Dunn, Carsten Grashoff

**Affiliations:** 1 Biophysics Program, Stanford University, Stanford, CA 94305 USA; 2 Group of Molecular Mechanotransduction, Max Planck Institute of Biochemistry, 82152 Martinsried, Germany; 3 Vegetative Anatomy, Institute of Anatomy, Faculty of Medicine, LMU Munich, 80336 Munich, Germany; 4 Department of Chemical Engineering, Stanford University, Stanford, CA 94305 USA; 5 Department of Quantitative Cell Biology, Institute of Molecular Cell Biology, University of Münster, 48149 Münster, Germany

## Abstract

Desmosomes are intercellular adhesion complexes that connect the intermediate filament cytoskeletons of neighboring cells, and are essential for the mechanical integrity of mammalian tissues. Mutations in desmosomal proteins cause severe human pathologies including epithelial blistering and heart muscle dysfunction. However, direct evidence for their load-bearing nature is lacking. Here we develop Förster resonance energy transfer (FRET)-based tension sensors to measure the forces experienced by desmoplakin, an obligate desmosomal protein that links the desmosomal plaque to intermediate filaments. Our experiments reveal that desmoplakin does not experience significant tension under most conditions, but instead becomes mechanically loaded when cells are exposed to external mechanical stresses. Stress-induced loading of desmoplakin is transient and sensitive to the magnitude and orientation of the applied tissue deformation, consistent with a stress absorbing function for desmosomes that is distinct from previously analyzed cell adhesion complexes.

## Abstract (web summary)

Desmosomes are intercellular adhesion complexes that connect the intermediate filament cytoskeletons of neighboring cells but direct evidence for their load-bearing nature is lacking. Here the authors develop FRET-based tension sensors to measure the forces experienced by desmoplakin and infer that desmosomes become mechanically loaded when cells are exposed to external mechanical stresses.

## Introduction

**Fig. 1:**
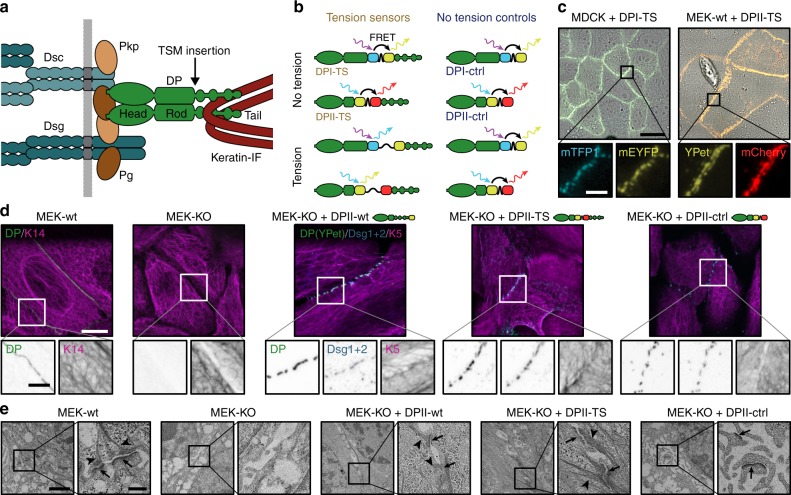
Desmoplakin (DP) tension sensors localize to desmosomes (DSMs). **a** DSM adhesion is mediated by the desmosomal cadherins desmocollin (Dsc) and desmoglein (Dsg), which engage the adapter proteins plakophilin (Pkp) and plakoglobin (Pg); DP forms the connection to intermediate filaments (IFs). The tension sensor module (TSM) was inserted into DP after the rod domain. **b** DP tension sensors (DPI-TS, DPII-TS) were generated along with controls lacking the IF-binding C-terminal region (DPI-ctrl, DPII-ctrl). The TSM comprises the F40 linker peptide (GPGGA)_8_ flanked by mTFP1/mEYFP in DPI, and YPet/mCherry in DPII. Tension reduces FRET in DP-TS but not in DP-ctrl constructs. **c** Live-cell imaging reveals normal subcellular localization of DPI-TS (expressed in MDCK cells) and DPII-TS (expressed in MEK-wt one day after DSM induction by Ca^2+^). Scale bar: 20 μm; in zoom: 4 μm. **d** DPII constructs localize to intercellular junctions in MEK-KO one day after DSM induction, similar to endogenous DP in MEK-wt. Immunostainings show that DPII constructs co-localize with Dsg1/2, and that full-length constructs but not DPII-ctrl mediate the recruitment of a coherent keratin 5 (K5) network. Images are summed projections of nine optical slices covering 3.1 μm. Scale bars: 10 μm; in zoom: 4 μm. **e** Ultrastructural analysis of MEKs one day after DSM induction reveals that the expression of DPII-wt and DPII-TS but not DPII-ctrl rescue both the DSM formation (arrows) and IF attachment (arrow heads) defects of MEK-KO. Electron microscopy images are contrast adjusted. Scale bars: 2 μm; zoom: 0.5 μm

Desmosomes (DSMs) are cadherin-mediated junctional complexes that mechanically couple the intermediate filament (IF) network of neighboring cells via protein complexes containing plakoglobin, plakophilins, and the IF-binding protein desmoplakin (DP), a large adapter molecule that provides an indispensable physical linkage between the inner desmosomal core and the IF network (Fig. 1a)^1–3^. Available evidence demonstrates an essential role for DSMs in maintaining the physical integrity of epithelia and heart muscle tissue during embryonic development and adult tissue homeostasis. In genetic mouse models, for instance, loss of DP causes early lethality prior to gastrulation, while skin-specific depletion leads to severe skin blistering upon mechanical stress and perinatal lethality^4,5^. In humans, mutations in desmosomal proteins or autoantibody-induced destabilization of DSMs cause skin-blistering diseases and are associated with severe cardiac defects such as arrhythmic cardiomyopathy^6–12^.

Even though DSMs are clearly essential for the integrity of many vertebrate tissues, the molecular details of force propagation across the DSM–IF junction are poorly understood. In fact, there is limited direct evidence for when and even whether the molecular components of DSMs bear mechanical loads in cells^13^. To address this knowledge gap, we applied our previously described FRET-based tension sensor module (TSM)^14–18^ to visualize and quantify piconewton (pN)-scale forces across DP (Fig. 1a). In addition, we leveraged the punctate geometry of DSMs to determine how tension on DP related to the magnitude and orientation of strain-induced tissue deformations. These experiments revealed that DP, and by extension DSMs, experienced negligible loads due to cell-generated forces, for example, during collective cell migration. Instead, our data demonstrated that DSMs came under load when cells were subjected to large, externally generated mechanical stresses that could threaten tissue integrity. In addition, we found that DSMs perform an unexpected role in supporting load in keratinocytes adhering to substrates of low stiffnesses. These observations provide a coherent framework for understanding how DSMs contribute to the construction of epithelia that are both dynamic and physically robust, two seemingly contradictory properties that are nonetheless essential for mammalian life.

We note that this work represents a collaboration that began as two independent studies of the two major DP splice isoforms, termed DPI and DPII. The coordination of our efforts led to a more robust and comprehensive understanding of DP function than would have been possible in either model system alone.

## Results

### Generation of isoform-specific desmoplakin tension sensors

DP consists of an N-terminal domain that binds to desmosomal cadherins, a C-terminal region that connects to IFs, and a central coiled-coil rod domain that mediates dimerization (Fig. 1a). The two major DP splice isoforms, DPI and DPII, are expressed in a tissue-specific manner and characterized by rod domains of different length^19^. We inserted the F40-based TSM, which responds to mechanical forces of about 1–6 pN^14,17^, into an unstructured region after the rod domain, prior to Pro1946 for DPI (called DPI-TS) and prior to Thr1354 for DPII (DPII-TS; Fig. 1b). This integration site was selected to facilitate the quantification of forces that are transduced from cell–cell junctions to the IF network, and minimizes potential interference with both DP dimerization and binding to either keratin filaments or junctional proteins. Control constructs incapable of bearing mechanical load, termed DPI-ctrl and DPII-ctrl, were engineered by removing the C-terminal keratin-binding domain (Fig. 1b). These truncated DP controls still localize to intercellular junctions^3,20,21^ but are unable to interact with keratin filaments and therefore control for potential confounding effects that could arise from local changes in the subcellular environment and modify FRET. As a control for protein functionality, we used constructs in which the DP molecule was fluorescently tagged at the C-terminus (DPI-wt and DPII-wt; Supplementary Fig. 1).

We expressed DPI-constructs in Madin Darby canine kidney (MDCK) cells, and DPII constructs in murine epidermal keratinocytes (MEKs) and analyzed cells by live-cell imaging and immunostaining. In both cases, a punctate recruitment of full-length DP constructs to cell–cell junctions was observed, matching the localization of the native protein (Fig. 1c and Supplementary Fig. 1a). To test whether DPI-TS and DPII-TS were properly recruited in the absence of endogenous DP, we transiently expressed DP sensors and controls in MEKs lacking DP (MEK-KO; Fig. 1d and Supplementary Fig. 1b, c), and observed that DPI-TS and DPII-TS localized to cell–cell contacts efficiently. Junctional puncta co-localized with the desmosomal cadherins desmoglein-1 and -2 (Dsg1/2), with plakophilin-1 (Pkp1), and plakoglobin (Pg), suggesting that the observed structures recapitulate bona fide DSMs (Fig. 1d and Supplementary Fig. 1d, e). The truncated constructs DPI-ctrl and DPII-ctrl also showed the expected subcellular localization to desmosomal puncta, consistent with a previously proposed role for DP in organizing desmosomal architecture independent of the C-terminal keratin-binding domain^5^. As expected^22^, we observed the recruitment of DP-constructs to MEK-KO intercellular junctions only after addition of sufficient Ca^2+^ to the cell-culture media. Ultrastructural analysis by electron microscopy revealed that wild-type MEKs (MEK-wt) formed the expected disc-shaped structures characterized by two distinct electron-dense layers at each side of a cell–cell contact that were connected to filamentous structures, presumably IFs^23^. Such complexes were not observed in MEK-KO and rarely detected in MEK-KO expressing DPII-ctrl, but efficiently induced by transient expression of DPII-wt and DPII-TS (Fig. 1e). These data suggested that TSM integration into DP preserved protein functionality and thereby the ability to rescue DSM formation.

### No tension across DPI under homeostatic conditions

**Fig. 2:**
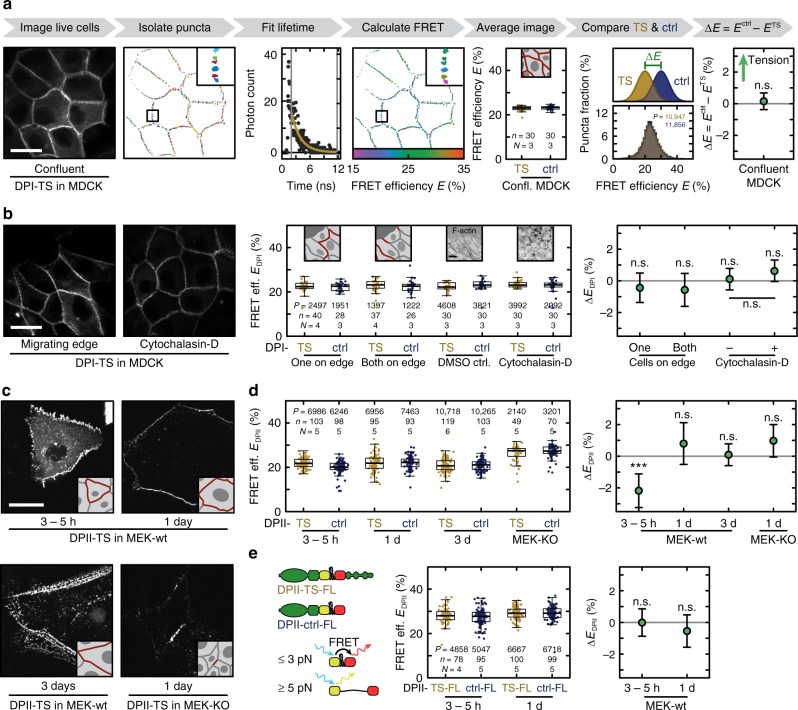
Desmoplakin tension is negligible under homeostatic conditions. **a** Donor intensity signals were masked and thresholded to generate a segmentation map of individual DSM puncta. For each punctum, a fluorescence lifetime was determined and the corresponding FRET efficiency calculated. FRET efficiencies for DPI-TS (yellow) and DPI-ctrl (blue) were indistinguishable in confluent MDCK monolayers. The median FRET efficiency per image is shown as a boxplot and reflects the underlying distributions of individual puncta values that were used to calculate the mean change in FRET efficiency as Δ*E* = *E*^ctrl^ − *E*^TS^. **b** No FRET efficiency differences were observed at the edge of migrating MDCK cell sheets in cell–cell junctions categorized as having one or both cells on the colony edge. Similarly, treatment of confluent MDCK monolayers with the actin-destabilizing drug cytochalasin-D (see Supplementary Fig. 2c for immuno-staining) did not induce a significant change in FRET efficiency. **c** DPII constructs were expressed in MEK-wt monolayers and imaged at distinct time points after DSM induction (3–5 h, 1 day, and 3 days). MEK-KO were imaged one day after DSM induction. **d** In all conditions, the mean changes between DPII-TS (yellow) and DPII-ctrl (blue) were consistent with little or no tension, with tension corresponding to Δ*E*_DPII_ > 0. **e** No FRET efficiency differences were observed between DPII-TS-FL (yellow) and DPII-ctrl-FL (blue). Scale bars: 20 μm. Boxplots show median, 25th and 75th percentile with whiskers reaching to the last data point within 1.5× interquartile range. Δ*E* is plotted as mean difference with 95% CI; lmer-test: ***p < 0.001, n.s. (not significant) *p* ≥ 0.05. Numbers of puncta (*P*), images (*n*), and independent experiments (*N*) are indicated in the figure. Source data are provided as a Source Data file

Previous results indicated a crucial role of cadherin–keratin interactions in orienting the collective migration of *Xenopus* mesendoderm cells^24,25^. We examined whether intercellular forces generated during collective cell migration of cultured cells might induce mechanical loads across the DSM–IF junction. To do so, we quantified DPI-TS and DPI-ctrl FRET efficiencies using fluorescence lifetime imaging microscopy (FLIM) in confluent MDCK monolayers (Fig. 2a), and in MDCK cells migrating to fill a gap in a confluent monolayer (Fig. 2b). We observed that DPI-TS FRET efficiencies were statistically indistinguishable from those measured for DPI-ctrl in both cases (Fig. 2a, b) indicating little or no tension across DPI in both confluent monolayers and at the edge of expanding monolayers. We next seeded MDCK cells expressing either DPI-TS or DPI-ctrl at different densities onto collagen-coated glass coverslips and analyzed FRET at DSMs. To ensure that we were not limited by the FLIM-FRET approach, which relies on extended image acquisition times, we performed ratiometric FRET measurements that do not yield an absolute FRET efficiency value but benefit from shorter acquisition times. Cell numbers were set to obtain colonies in which virtually all cells were on an open edge boundary (sparse), cells formed larger colonies with free edges (sub-confluent), or cells formed monolayers (confluent). Despite large differences in cell spread area, we measured no significant change in average FRET index relative to the truncated control in sparse, sub-confluent, and confluent monolayers (Supplementary Fig. 2a). We further examined with FLIM the role of actomyosin contractility in DPI tension using the actin-destabilizing drug cytochalasin-D (Fig. 2b) and the ROCK inhibitor Y-27632 (Supplementary Fig. 2b). Again, we did not observe significant changes in FRET efficiency relative to control samples, despite clear effects of the drug treatments on the actomyosin network (Supplementary Fig. 2b, c). Finally, we treated DPI-TS and DPI-ctrl expressing cells with okadaic acid to induce a rapid collapse of keratin networks^26^, but did not observe any significant change in FRET efficiencies relative to control conditions (Supplementary Fig. 2d). All these findings led us to conclude that DPI experiences little or no tension in MDCK monolayers due to internal, cytoskeleton-generated forces.

### No tension across DPII under homeostatic conditions

Evidence from cell-culture experiments indicated a key role for the DPII isoform in keratinocyte DSM homeostasis^27^. To study DPII mechanics, we transiently expressed DPII constructs in MEK-wt and used live-cell FLIM to measure FRET efficiencies at different time points after induction of DSM formation by addition of Ca^2+^. In parallel, we performed a range of control experiments to validate lifetime determination, ensure data reproducibility, and confirm that effects of photobleaching were identical between DPII-TS and DPII-ctrl (Supplementary Fig. 3a‒c). We also generated a range of control constructs to identify suitable FRET donor-only controls for DPII experiments (Supplementary Fig. 3d‒f), and we confirmed that the contribution of intermolecular FRET was identical in DPII-TS and DPII-ctrl measurements (Supplementary Fig. 3g). Comparable to the DPI results described above, we observed FRET efficiencies for DPII-TS that were very similar to those for the DPII-ctrl construct (Fig. 2d). FRET efficiencies in DPII-TS cells were slightly elevated at early time points (3–5 h after Ca^2+^ addition) when compared to the control construct (Fig. 2d), which may reflect subtle differences in DSM architecture that influence fluorophore orientation, or alternatively transient compression of the F40 peptide^28,29^. No significant differences compared to control constructs were observed one and three days after DSM assembly, indicating a negligible level of tension across DPII in established DSMs (Fig. 2c, d). To determine whether unlabelled, endogenous DP affects the tension measurement, we expressed DPII constructs in MEK-KO cells. Yet again, FRET efficiencies for DPII-TS were similar to those for DPII-ctrl, suggesting that DPII does not experience significant loads under these conditions (Fig. 2c, d).

To validate these data sets, we generated DPII-TS and DPII-ctrl constructs based on the recently developed FL-TSM^17^. This sensor module uses a structurally different mechanosensitive peptide but also responds to low forces of about 3–5 pN. The analysis of the FL-TSM-based DPII constructs confirmed the lack of DP tension in MEKs at 3–5 h and one day after DSM assembly (Fig. 2e). Together, the data strongly suggest that homeostatic processes in cell culture such as collective cell migration in MDCK cells, changes in cell density, and DSM formation in keratinocytes do not involve mechanical loading of DP.

### DSMs are mechanically loaded in response to external stress

**Fig. 3:**
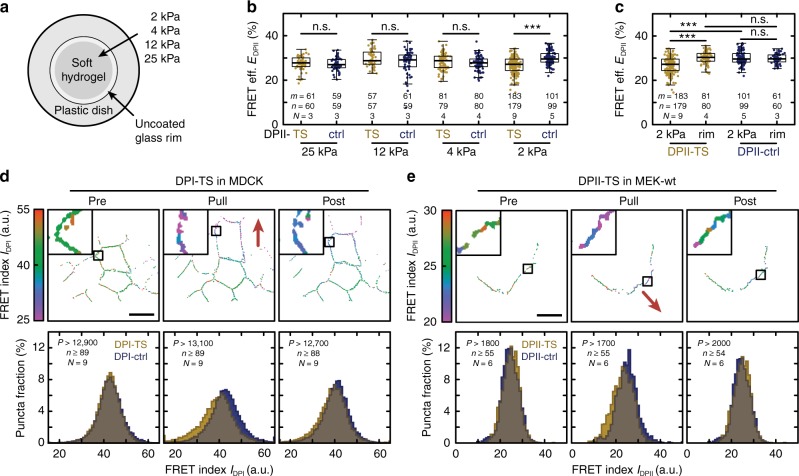
DP is mechanically loaded on soft hydrogels and in response to external force. **a** Hydrogels of defined stiffness were located in the center of a glass-bottom microscopy dish. **b** No FRET efficiency differences between DPII-TS (yellow) and DPII-ctrl (blue) were observed for 25, 12, and 4 kPa substrates, but DPII-TS showed a significant decrease in FRET efficiency for 2 kPa substrates. **c** The FRET efficiency decrease for DPII-TS on 2 kPa substrates is specific to cells cultured on the hydrogel. FRET efficiencies in cells on the uncoated glass rim surrounding the hydrogel were indistinguishable for DPII-TS and DPII-ctrl. **d** Top row: Example FRET index measurements for individual puncta of DPI-TS in an MDCK monolayer before (Pre), during (Pull), and after (Post) the application of external mechanical stress using a glass micropipette (the red arrow indicates pulling direction). Bottom row: FRET indices of DPI-TS (yellow) and DPI-ctrl (blue) were indistinguishable before pulling, but decreased specifically for DPI-TS during force application; the DPI-TS decrease partially recovered after relaxation. **e** Top row: Example FRET index measurements for DPII-TS expressed in a monolayer of MEK-wt in the course of a pulling experiment. Bottom row: Per-punctum FRET indices for cells expressing DPII-TS (yellow) and DPII-ctrl (blue) in confluent sheets of MEK-wt one day after DSM induction were indistinguishable before pulling. DPII-TS FRET indices were specifically reduced during pulling and returned to pre-pull levels after relaxation. **b**, **c** FRET efficiencies were determined via bulk fits. Numbers of manual masks (*m*), images (*n*), and independent experiments (*N*) are indicated in the figure. Boxplots show median, 25th and 75th percentile with whiskers reaching to the last data point within 1.5× interquartile range. Kolmogorov–Smirnov test: ***p < 0.001, n.s. (not significant) *p* ≥ 0.05. **d**, **e** Numbers of puncta (P), images (n), and independent experiments (N) are indicated in the figure. Scale bars: 20 μm. Source data are provided as a Source Data file

Given the importance of DP in vivo and its probable role in maintaining tissue integrity^5–10^, we next considered whether DP might experience loads in cells adhering to substrates that recapitulate physiologically relevant rigidities. As epidermal stiffness depends on the skin area and varies between epidermal layers^30,31^, we seeded DPII-TS or DPII-ctrl expressing MEKs onto different hydrogels characterized by Young’s moduli of 2, 4, 12 and 25 kPa, respectively (Fig. 3a, b), and performed live-cell FLIM experiments as described above. Consistent with the experiments on glass coverslips, no differences between DPII-TS and control constructs were detected on hydrogels of 4–25 kPa. However, DPII-TS FRET was significantly reduced on very soft 2 kPa substrates (Fig. 3b). To confirm the specificity of this observation, we measured cells not only at the center of the dish, where the substrate stiffness is 2 kPa, but also at the outer rim, where the soft hydrogel is missing and stiffness is high. Indeed, FRET levels were decreased only in DPII-TS cells that adhered at the center of the dish, while FRET values of control cells were insensitive to location (Fig. 3c). Together, these experiments demonstrated that DPII is not loaded in cells adhering to extracellular substrates that reflect a range of physiologically relevant rigidities but instead experiences tension in very soft environments.

We next tested whether externally applied forces might induce tension on DP. To apply mechanical stress directly to cell–cell junctions, we seeded MDCK cells or MEKs expressing the various DP-constructs onto coverslips, allowed sufficient time for the formation of DSMs, and then used a glass micropipette to pull on a localized area of the cell monolayer (Supplementary Fig. 4a, b and Supplementary Movie 1). We acquired ratiometric FRET measurements for cells in the undisturbed confluent layer before contact (Pre), at the pull maximum (Pull), and after relaxation by tip withdrawal (Post). To ensure reproducibility, we performed these experiments in nine (DPI) and six (DPII) different experimental preparations analyzing >12,000 desmosomal puncta (≥88 images) and >1700 desmosomal puncta (≥54 images), respectively. Data analysis algorithms were adjusted to exclude data from potentially overstretched and mechanically damaged cells, and we only considered junctional puncta that recoiled by at least 1 μm after relaxation. Strikingly, these experiments indicated that both DPI and DPII came under mechanical load upon stress application (Fig. 3d, e). Consistent with the FLIM measurements of MDCK monolayers described above (Fig. 2a), DPI-TS FRET index values were not different from those for DPI-ctrl before pulling. During pulling, an enrichment of low-FRET puncta was observed for DPI-TS relative to DPI-ctrl (Fig. 3d). Upon removal of the glass micropipette, junctions partially recoiled, and FRET values underwent a partial return to pre-pull values. Similarly, in MEK monolayers, the difference between DPII-TS and DPII-ctrl FRET index values was not significant before pulling, increased during force application, and relaxed after micropipette tip withdrawal (Fig. 3e). The specificity of this effect for DP-TS vs. DP-ctrl constructs strongly suggested that both DP isoforms become mechanically loaded in response to external stress. However, the moderate differences in average FRET for these puncta indicated that even under external stress only a subset of DP molecules experiences mechanical loads.

### Magnitude and orientation of stress determine DSM loading

**Fig. 4:**
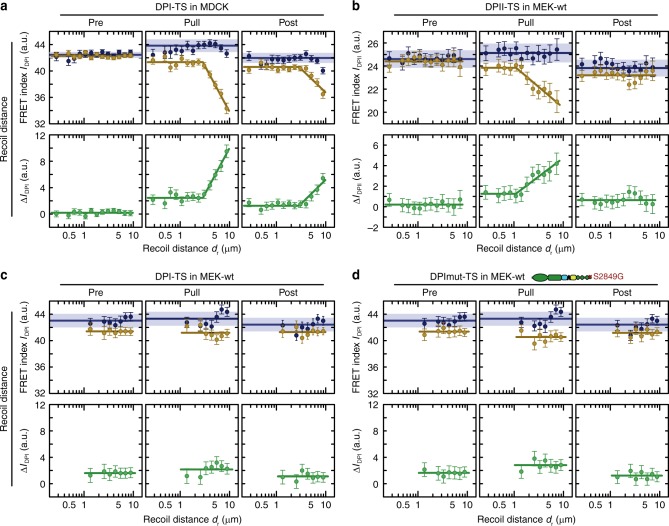
DP loading is sensitive to the magnitude of the applied deformation. **a** Before pulling (Pre), the FRET index difference (green) between DPI-TS (yellow) and DPI-ctrl (blue) in MDCK was close to zero, confirming that no tension was transmitted across DPI without external stress. During pulling (Pull), tension increased with deformation. After pipette tip withdrawal (Post), tension partially relaxed. Evidence of residual tension was observed at higher recoil distances. Data are from the pulling experiment represented in Fig. 3d. **b** Mechanical tension on DPII depends on the degree of deformation, and relaxes after external stress is released. Data are from the pulling experiment represented in Fig. 3e. **c** FRET index change for DPI-TS in MEKs was small in magnitude, even at high recoil distances. *P* > 1450 total puncta from *n* ≥ 36 images measured in six independent experiments. **d** Introduction of the S2849G mutation in DPI-TS does not alter sensitivity to recoil distance in MEKs. *P* > 1250 total puncta from *n* ≥ 35 images measured in six independent experiments. DPI-ctrl data were the same as in **c**. **a**–**d** Puncta with recoil distances *d*_r_ ≤ 10 μm were analyzed. The mean FRET index of DP-TS and DP-ctrl as well as the mean difference between DP-TS and the mean of all DP-ctrl puncta were calculated using the lmer-test and displayed with the 68% CI. Blue lines and corresponding shading indicate the mean with 95% CI of all DP-ctrl puncta. Yellow and green lines are guides to the eye. Source data are provided as a Source Data file

To evaluate this effect in more detail, we examined how changes in DP tension correlated with the magnitude and orientation of applied deformation within the cell monolayer. To this end, we determined the recoil distance *d*_r_ for DPI and DPII puncta and tested how the observed FRET difference between DP-ctrl and DP-TS (defined as ${\Delta I}_{{\mathrm{DP}}} = I_{{\mathrm{DP}}}^{{\mathrm{ctrl}}} - I_{{\mathrm{DP}}}^{{\mathrm{TS}}}$) correlated with this measure of deformation (Supplementary Fig. 4c). As expected, there was no significant correlation for Δ*I*_DP_ measured before pulling and the subsequent recoil distance, but Δ*I*_DP_ during pulling increased markedly with larger recoil distances for both DP isoforms. Post-pull data from MDCK-DPI experiments revealed that cells, especially those exposed to larger strains, maintained some residual DPI tension levels (Fig. 4a). In MEK-DPII experiments post-stress values of Δ*I*_DP_ were statistically indistinguishable from pre-pull values, suggesting that DPII bore forces transiently in MEKs and returned to the low-tension state upon removal of external forces (Fig. 4b). To determine whether the observed differences in DPI and DPII experiments could be explained by differences in cell type, we expressed the DPI-constructs in MEKs and repeated the FRET pulling experiments. Intriguingly, the increase in Δ*I*_DP_ during pulling was less pronounced compared to that observed in DPII experiments (Fig. 4c). Even the insertion of the S2849G mutation into DPI, which is expected to enhance DP coupling with IFs^32^, did not increase DPI tension (Fig. 4d). These observations lend support for a previously suggested DP-isoform-specific function in keratinocytes^27^.

**Fig. 5:**
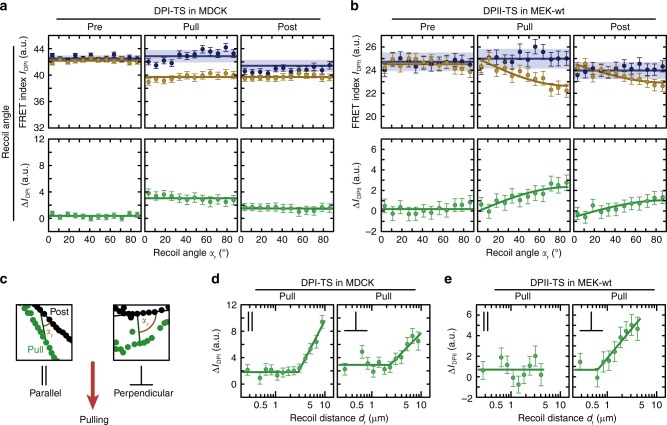
DPII loading is sensitive to the orientation and magnitude of the applied deformation. **a** Tension across DPI in MDCK cells did not depend on the recoil angle. Data are from the pulling experiment represented in Fig. 3d. **b** In MEK-wt, DPII tension increased for increasing recoil angles. Data are from the pulling experiment represented in Fig. 3e. **c** Puncta were classified as parallel (*α*_r_ ≤ 45°) or perpendicular (*α*_r_ > 45°) to pulling direction. **d** The effect of magnitude and orientation of the external deformation showed no apparent additive effect for DPI-TS in MDCK monolayers. **e** The effect of magnitude and orientation of the external deformation were additive for DPII-TS in MEK-wt. **a**–**e** The mean FRET index of DP-TS and DP-ctrl as well as the mean difference between DP-TS and the mean of all DP-ctrl puncta were calculated using the lmer-test and displayed with the 68% CI. Blue lines and corresponding shading indicate the mean with 95% CI of all DP-ctrl puncta. Yellow and green lines are guides to the eye. Source data are provided as a Source Data file

Finally, we considered how the orientation of applied stress affected DP loading. We defined the recoil angles *α*_r_ such that desmosomal puncta that relaxed along the direction of the cell–cell junction were assigned a recoil angle of *α*_r_ = 0°, whereas puncta recoiling perpendicular to the cell–cell junction were set to *α*_r_ = 90°. We then plotted FRET differences of the pre, pull, and post state according to the recoil angle *α*_r_ (Fig. 5a, b). No significant correlation was observed in MDCK monolayers expressing DPI-TS (Fig. 5a), possibly because deformations propagated across 4–5 cells in these monolayers, a range over which local stress anisotropies might be expected to decay (Supplementary Movie 1). In DPII-TS MEK experiments, where deformations propagated over shorter distances, FRET differences were larger for high recoil angles relative to low recoil angles (Fig. 5b). Analyzing the interdependence of these observations confirmed that the angular dependency of DPII loading in MEKs was strain-sensitive: DPII was exposed to mechanical forces specifically in those junctions that experienced perpendicular deformations and large recoil distances (Fig. 5c–e). These observations suggest that stress propagation through the IF cytoskeleton can be highly anisotropic at the level of single cells, a finding consistent with a proposed role for the IF cytoskeleton in controlling collective cell migration^24,25^.

## Discussion

The data presented here demonstrate that DSMs are functionally distinct from previously described cellular adhesion complexes such as the adherens junction that bear mechanical tension under steady-state conditions^33,34^. While these actin-associated adhesions experience tension due to motor protein contractility, the DSM appears to experience little or no load resulting from contractile forces generated by individual cells if the substrate stiffness is greater than ~2 kPa. Instead, the DSM–IF junction comes under tension in response to externally applied mechanical deformations, suggesting that the DSM is specialized to function as a stress-absorbing adhesion complex. This interpretation is consistent with the phenotypes caused by epidermis-specific deletion of DP in mice^5^, which undergo normal development but show severe epidermal blistering after birth. Our observations thus contribute to a better molecular understanding of severe epidermal diseases of human patients resulting from mutations in desmosome-associated proteins which are characterized by massive epidermal defects in response to mechanical stress^6–10,35^.

The finding that, for cells on stiff substrates, tension is negligible during homeostatic processes such as cell migration and changes in cell density is consistent with the pronounced elasticity of IF networks at low strain^36,37^, and the fact that these networks can stiffen under deformation^38–40^. Similarly, the observation that DSMs become mechanically engaged in response to external stresses suggests a mechanical role for DSMs in responding to deviations in cell shape that occur on faster time scales (seconds–minutes) than are typically accommodated during cell-generated shape change (minutes–hours). Clarifying the interdependent nature of deformation magnitude, strain rate, and molecular stress experienced by DSMs is a promising avenue for future research, for which sensors such as those described here may be particularly useful.

Intriguingly, our measurements indicate that DPII experiences tension in keratinocytes adhering to very soft 2 kPa substrates. We speculate that this observation may reflect long-range propagation of tension through the IF cytoskeletons of neighboring cells, analogous to intercellular stress propagation across the actin cytoskeletons of epithelial cells^41^. Such an effect might be expected to occur specifically under circumstances in which substrate stiffness is similar to that of the IF network, reported to be <1 kPa^42^. Intercellular tension transmission through the IF cytoskeleton would be consistent with the reported role for IFs in coordinating collective cell migration in the *Xenopus* mesendoderm^24^. Further insight into where and when the IF cytoskeleton has an active role in shaping tissue mechanics, for example during embryogenesis, represents a fascinating question for future investigations.

It is interesting to note that we obtained very similar but not identical results in two cellular systems: MDCK cells express keratins (K)8 and K18, which are found in simple epithelia, whereas MEKs are characterized by K5/K14 networks typical for basal keratinocytes. Thus, the impact of distinct keratin networks on DSM mechanics should be investigated in the future, and it may be especially interesting to explore the mechanical role of DSMs in heart muscle cells, which experience a very different mechanical environment and engage the IF desmin. Our data support a DP-isoform-specific function in keratinocytes, as proposed earlier^27^ and consistent with the observation that DPII is oriented perpendicular to the cell–cell contact^43^. Only DPII displayed strong distance and angle-dependent loading in these cells, an effect that should be studied in more detail. Finally, IF networks are known to undergo stress-dependent remodeling^44^. Future measurements of DP tension in the setting of mutations that alter IF remodeling will help to build a better understanding of how DSMs and the IF cytoskeleton respond to mechanical load.

While this paper was under review, a separate study was published indicating that desmoglein-2 experienced mechanical load in unstressed MDCK cells^45^. Our measurements show negligible tension on DP under similar conditions. An alternative connection between desmosomal cadherins and the actin cytoskeleton is one possible explanation for these apparently contrasting observations. Future studies, potentially targeting other desmosomal components, may help to shed light on when and how desmosomal cadherins experience mechanical load.

Altogether, our data suggest that DSM–IF junctions are tuned to withstand external mechanical stresses, but can do so without hindering the cellular movements and shape changes that are essential to maintaining tissue homeostasis. This physical role is distinct from those of other intercellular adhesion complexes^15,46^, and can help explain how the dynamics of DSMs are tuned to allow the construction, maintenance, and repair of tissues that are exposed to high external stresses.

## Methods

### Antibodies

The following primary antibodies were used: mouse anti-desmoplakin I/II (Abcam, ab16434; dilution: 1:100), rabbit anti-keratin-5 (BioLegend, 905501; 1:1000), rabbit anti-keratin-14 (BioLegend, 905301; 1:1000), mouse anti-desmoglein-1/2 (Progen Biotechnik, 61002; 1:200), mouse anti-plakophilin-1 (Santa Cruz, sc-33636; 1:200), mouse anti-plakoglobin (Thermo Fisher Scientific, 13-8500; 1:200), mouse anti-keratin-18 (Thermo Fisher Scientific, MA1-06326; 1:100), and mouse anti-phospho-myosin light chain 2 (Cell Signaling Technology, 3675; 1:200). The following secondary antibodies were used: anti-mouse IgG Alexa Fluor-647 (Thermo Fisher Scientific, A31571; 1:500), anti-mouse IgG Alexa Fluor-488 (Thermo Fisher Scientific, A21200; 1:500), anti-rabbit IgG Alexa Fluor-405 (Thermo Fisher Scientific, A31556; 1:500), and anti-mouse IgG Alexa Fluor-647 (Cell Signaling Technology, 4410; 1:1000).

### Construct generation

For DPI-constructs, human DPI-GFP cDNA (Addgene, 32227) was used as a template. DPI-TS was assembled by a custom cloning service (Epoch Life Science, Inc). The mTFP1-F40-mEYFP module was inserted prior to Pro1946 and flanked by 25 and 24 amino acid (aa) long flexible linker sequences (N-term: LIKGSGGTGSTSGGSGGSTGGGTGA, C-term: GTGGGTSGGSGGSTSGTGGSGSGR), and cloned into the pSBtet-Pur plasmid downstream of the TRE promoter (Addgene, 60507). The DPI-constructs were also inserted into the piggyBac vector downstream of the hEF1α promoter (DNA 2.0, pJ509-02) for transient expression in MEKs. The photometric control was generated with the QuickChange Lightning Site-Directed Mutagenesis Kit (Agilent), replacing the essential tyrosine for chromophore formation with a glycine (Y67G for mEYFP)^47^. DPII was generated by overlap extension polymerase chain reaction (PCR) using DPI as a template (overlap region: 5′-GAA TAT GAA AAT GAG CTG GCA AAG GCA TCT AAT AGG ATT CAG GAA TCA AAG-3′). The YPet-F40-mCherry module (Addgene, 101252) or YPet-FL-mCherry module (Addgene, 101170) were inserted prior to Thr1354 and flanked by short linkers (N-term: VE, C-term: AAA). DPII constructs were either assembled by standard enzyme-based cloning techniques as described previously^48^ or with Gibson assembly (New England Biolabs (NEB), E2621L). Donor-only controls were generated by Gibson assembly and used TagBFP (Evrogen), SNAP (NEB, N9183S), and fluorescently dead mCherry(Y72L)^49^. Final DPII expression constructs were inserted into pLPCX (Clontech) after the CMV promoter; DNA sequencing (Eurofins Genomics) confirmed the correct base pair sequence of these constructs. Plasmids encoding all relevant sensor and control constructs are available on Addgene (DPI, 119186-119188, 119190 and DPII, 118714-118724).

### Cell culture and construct expression

MDCK II cells (Sigma, 00062107) were maintained in low-glucose DMEM (Thermo Fisher Scientific, 11885-076), supplemented with 10% fetal bovine serum (FBS, Corning, 35011CV) and 1% penicillin–streptomycin (Thermo Fisher Scientific, 15140122). Transfection was performed with a Lonza 4D Nucleofector using the SE Cell Line solution (Lonza, V4XC-1012) and protocol CM-113. For each transfection, 1.8 μg cDNA of the expression construct and 0.2 μg of the transposase plasmid were used (Addgene, 34879). After 3 days, cells were selected in growth medium containing 2.5 μg mL^−1^ of puromycin (Thermo Fisher Scientific, A1113803). Fluorescence activated cell sorting (FACS) was used to enrich for DPI-TS and DPI-ctrl expressing cells (Stanford FACS facility), and sorted cells were cultured for 14 days in the absence of doxycycline to dilute out the expression of exogenous DPI-constructs prior to making frozen stocks. Parental and FACS-sorted cells were free of mycoplasma contamination (PromoKine, PK-CA91-1096).

For imaging experiments, MDCK cell lines were treated with 0.1 μg mL^−1^ (DPI-ctrl) or 0.5 μg mL^−1^ (DPI-TS and photometric controls) doxycycline in order to achieve similar levels of construct expression for the cell lines, and plated onto collagen-coated coverslips (Cellvis, D35-20-1.5-N) 48 hours (h) prior to imaging. About 24 h before imaging, the cell-culture medium was exchanged for imaging medium composed of L-15 media (Thermo Fisher Scientific, 21083027) supplemented with 1% FBS, 1% penicillin–streptomycin, 1% ITS-A (Thermo Fisher Scientific, 51300044), and the respective concentration of doxycycline.

MEK-wt and desmoplakin-deficient MEK-KO cells were a gift from Dr. Kathleen Green (Northwestern University). Cells were free of mycoplasma contamination (Jena Bioscience, PP-401L) and maintained in the fully defined, animal-component free culture medium CnT-Prime (Cell-N-Tec), which contains 0.07 mM CaCl_2_. Cells were split with accutase (Cell-N-Tec, CnT-Accutase-100) and sub-cultured at a splitting ratio of 1:3–1:5. cDNA constructs were transiently transfected with Lipofectamine 3000 (Thermo Fisher Scientific, L3000015). For FRET measurements, MEKs were seeded in live-cell imaging dishes (Ibidi, 81158) or imaging dishes with a hydrogel of defined stiffness (Matrigen, SV3520-EC-2/4/12/25 PK) and transfected with 4.5 μg cDNA and 7.5 μL Lipofectamine. For immunostainings, cells were seeded on no. 1.5 glass slides (Menzel) and transfected with 1 μg cDNA using 2 μL Lipofectamine. To induce DSM formation in MEKs, media was exchanged to CnT-Prime supplemented with 1% penicillin–streptomycin and 1.5 mM CaCl_2_.

### Drug treatments

For actomyosin inhibitor treatments, monolayers were prepared in a collagen-coated multi-well plate (Cellvis, P24-1.5H-N). Y-27632 (Sigma, Y0503) was used at a concentration of 10 μM for 60 minutes (min), cytochalasin-D (Sigma, C2618) at a concentration of 1.5 μM for 30 min, and okadaic acid (Cayman Chemical, 10011490) was used at a concentration of 50 nM for 12 h; an equivalent volume and time of the corresponding drug solvent was used as control for Y-27632 (water; 1:500), cytochalasin-D (DMSO; 1:3333), and okadaic acid (ethanol; 1:2500).

### Immunostaining

During all procedures, samples were protected from light to prevent photobleaching of the expressed fluorescent proteins. For F-actin and p-MLC immunostaining, MDCK cells were fixed with 4% paraformaldehyde in phosphate buffered saline (PBS, pH 7.4). For all other immunostainings of MDCK cells and MEKs expressing DPI-constructs, samples were fixed with pre-cooled methanol at −20 °C for 8 min and washed twice with PBS (pH 7.4). Blocking was performed at room temperature (RT) for 1 h in PBS containing 1% bovine serum albumin (BSA) and 0.1% Triton X-100 (Sigma). Primary antibodies (i.e., anti-desmoplakin I/II, anti-keratin-18, anti-keratin-5) were diluted in blocking buffer and incubated with cells for 1–2 h at RT. Secondary antibodies were diluted in blocking buffer containing 1 μg mL^−1^ Hoechst 34580 (Thermo Fisher Scientific, H21486) and incubated for 1–3 h with cells at RT. For labelling F-actin networks, ActinRed (Thermo Fisher Scientific, R37112) was added to the secondary antibody buffer.

For immunostaining MEKs expressing DPII constructs, cells were rinsed twice with PBS, the cytoskeleton stabilizing buffer (1 mM EGTA, 1 mM MgCl_2_, 50 mM glycerol, 25 mM PIPES, pH 7.4), and fixed for 8 min with pre-cooled methanol at −20 °C; cells were washed once with PBS, then PBS containing 0.02% Tween-20 (PBST), and PBST containing 1% BSA (PBSTB). Next, primary antibodies were diluted in PBS containing 3% BSA and incubated with cells overnight at 4 °C. The next day, cells were washed with PBST and PBSTB, and incubated for 1 h at RT with secondary antibodies. Finally, cells were washed with PBST and PBS and mounted in Prolong Gold antifade mounting solution (Thermo Fisher Scientific, P36934). Immunostainings were imaged using a Zeiss LSM 780 confocal microscope.

### Transmission electron microscopy (TEM)

Transfected cells were fixed with 1% glutaraldehyde for 1 h at 37 °C, followed by three times washing with PBS and subsequent incubation with 2% osmiumtetroxide solution for 1 h at 4 °C. Afterwards, samples were dehydrated through an ethanol series from 20 to 100% and embedded with Epon for 24 h at 80 °C. Finally, ultrathin sections (60–80 nm) were cut with a diamond knife and stained with a saturated solution of uranyl acetate for 40 min and lead citrate for 5 min. Images were acquired with the transmission electron microscope Libra 120 (Zeiss).

### Fluorescence lifetime imaging microscopy (FLIM)

For DPI-TS experiments, fluorescence lifetime data were collected using a Zeiss LSM 780 confocal microscope equipped with two-photon pulsed excitation MaiTai Ti:Sapphire laser tuned to 860 nm, a Becker & Hickl SPC-150 detection system, a LCI Plan Apo ×40 water immersion objective, a band-pass filter (Semrock, 475/28 nm) for mTFP1, and a heating chamber (37 °C). Images were acquired as 512 × 512 pixels covering 70.85 × 70.85 μm^2^; for each experimental condition, 10 images were taken on 3–4 preparations resulting in a total of about 25–40 images. FLIM experiments for DPII constructs in MEKs were performed on a confocal laser scanning microscope (Leica TCS SP5 X) equipped with a pulsed white light laser (NKT Photonics), a FLIM X16 TCSPC detector (LaVision Biotech), a ×63 water objective (HCX PL APO CS), a band-pass filter (Chroma, 545/30 nm) for YPet, and a heating chamber (37 °C, 5% CO_2_; Ibidi). Cells on hydrogel dishes were imaged at RT. Images were acquired with a scanning velocity of 400 Hz for 512 × 512 pixels covering 61.51 × 61.51 μm^2^. For each experimental condition, 15–20 images were taken on 3–8 individual days resulting in a total of about 50–100 images.

### FLIM-FRET analysis

To analyze time-correlated single photon counting (TCSPC)-FLIM data, custom-written MATLAB programs and software based on previously published algorithms were used^17^. For measurements in MEKs, cell–cell junctions were extracted by manually drawing masks. Next, the desmosomal signal was isolated with a binary mask generated from the intensity image by blurring the image (Gaussian, *σ* = 3 pixels) and isolating connected bright regions. For bulk fits, all photons detected within the resulting mask were used to determine the fluorescence lifetime. For individual puncta, the masked intensity image was blurred (Gaussian, *σ* = 3 pixels) and pixels were assigned to the nearest local intensity maximum. Regions smaller than nine pixels were excluded.

For measurements in MDCK cells, a segmentation map was generated from the intensity image by selecting pixels brighter than one standard deviation above the mean image pixel intensity after band-pass filtering to enhance features of interest (3–10 pixel diameter). To isolate individual puncta, pixels were assigned to the nearest local intensity maximum, and regions smaller than nine pixels were excluded. Furthermore, puncta in the cytosol or in regions with abnormally high autofluorescence (i.e., from cell debris immediately above the cell monolayer) were manually excluded.

To determine the fluorescence lifetime, an exponential decay was fitted to the summed photon count time trace from each mask or punctum. The fit to the decay curve was set to start 0.56 ns after the maximal photon count to minimize the contribution of the instrument response function and autofluorescence. Fitting was performed using MATLAB’s ‘fmincon’ with a maximum-likelihood cost function based on Poisson statistics^50,51^.

The FRET efficiency *E* was calculated from the lifetime of the donor in presence of an acceptor *τ*_DA_ and the mean donor-only lifetime $\overline {{\mathrm{\tau }}_{\mathrm{D}}}$, according to Eq. (1):

1 $$E = 1 - \frac{{{\mathrm{\tau }}_{{\mathrm{DA}}}}}{{\overline {{\mathrm{\tau }}_{\mathrm{D}}} }}.$$

The mean donor-only lifetime was determined from matched constructs with disrupted chromophore formation of the acceptor (mCherry-Y72L and EYFP-Y67G; Supplementary Fig. 3d-f). FRET efficiencies were calculated with 2.94 ns for YPet-F40-mCherry (Supplementary Fig. 3f), 2.98 ns for YPet-F40-mCherry on soft substrates and YPet-FL-mCherry (Supplementary Fig. 3h, i), 2.52 ns for mTFP1-F40-mEYFP, and 2.55 ns for mTFP1-F40-mEYFP in drug treatment experiments. The minimal required photon number was determined by the reduction in spread of the lifetime fits to be 1000 photons for DPII, 175 photons for DPI, and 275 photons for DPI in drug treatment experiments. Individual puncta fits with extreme (<0% or >100%) values were excluded from the analysis. No fits were excluded from bulk analyses.

### Sensitized emission ratiometric FRET analysis

Epifluorescence imaging was performed on an inverted Nikon Ti-E microscope controlled with Micromanager 1.4.22^52^. The microscope was equipped with a Heliophor light engine (89 North), an Andor sCMOS Neo camera using a CFI Plan Apo Lambda ×40 air objective lens and a heating chamber (37 °C). Sensitized emission FRET (SE-FRET) analysis was performed with three channel acquisitions on an epifluorescence microscope: donor signal after donor excitation (*D*_obs_), acceptor signal after donor excitation (*F*_obs_), and acceptor signal after acceptor excitation (*A*_obs_). Images were flat-field corrected by subtracting dark frame intensity and dividing by a normalized calibration image from a uniformly fluorescent sample in PBS (riboflavin for CFP, FRET, and YFP channels, and TRITC for RFP). A rolling ball background filter (50 pixel/~8 μm diameter) was employed to remove background intensity from each channel. After flat-field correction and background subtraction, the corrected FRET intensity (*F*_cor_) was determined by linear de-mixing based on correction factors *ε* (Eq. 2). The values for *ε* were obtained by fitting fluorophore-only samples^53^.

2 $$\left[ {\begin{array}{*{20}{c}} {D_{{\mathrm{cor}}}} \\ {F_{{\mathrm{cor}}}} \\ {A_{{\mathrm{cor}}}} \end{array}} \right] = \left[ {\begin{array}{*{20}{c}} 1 & {\varepsilon _{{\mathrm{FD}}}} & {\varepsilon _{{\mathrm{AD}}}} \\ {\varepsilon _{{\mathrm{DF}}}} & 1 & {\varepsilon _{{\mathrm{AF}}}} \\ {\varepsilon _{{\mathrm{DA}}}} & {\varepsilon _{{\mathrm{FA}}}} & 1 \end{array}} \right]\left[ {\begin{array}{*{20}{c}} {D_{{\mathrm{obs}}}} \\ {F_{{\mathrm{obs}}}} \\ {A_{{\mathrm{obs}}}} \end{array}} \right]$$

For the mTFP1/mEYFP FRET pair used in DPI-constructs the correction factors were *ε*_FD_ = 4.9 · 10^−3^, *ε*_AD_ = 4.5 · 10^−4^, *ε*_DF_ = 0.62, ε_AF_ = 0.092, *ε*_DA_ = 2.4 · 10^−3^, and *ε*_FA_ = 3.8 · 10^−3^. For the YPet/mCherry FRET pair used in DPII constructs the correction factors were *ε*_FD_ = 2.4 · 10^−3^, *ε*_AD_ = 1.3 · 10^−4^, *ε*_DF_ = 0.29, *ε*_AF_ = 0.056, *ε*_DA_ = 0.038, and *ε*_FA_ = 0.13. A segmentation map was generated from the acceptor channel (*A*_cor_) by selecting pixels brighter than four standard deviation above the mean pixel intensity of a background region of the monolayer and using the same puncta-based signal extraction method as with the FLIM-FRET analysis. The FRET index I was calculated by Eq. (3):

3 $$I = \frac{{F_{{\mathrm{cor}}}}}{{D_{{\mathrm{cor}}} + F_{{\mathrm{cor}}}}} \cdot 100.$$

Puncta that were too dim (<5000 total intensity [a.u.] in *A*_cor_ for DPI-TS, or <2000 total intensity [a.u.] in *A*_cor_ for DPII-TS) or that had extreme FRET index values (<0 or >100) were excluded; puncta in regions showing misalignment in an overlay of donor and acceptor channel acquisition images were manually discarded.

### Micromanipulation experiment and recoil analysis

A glass micropipette was mounted on a micromanipulation device assembled with two manual single axis stages (ThorLabs, PT1) for *x*–*y* translation and a motorized single axis stage (ThorLabs, MTS50) to control height. The micropipette was lowered onto the confluent monolayer until a cell was pinched between the micropipette and the glass surface, and was then pulled horizontally. The monolayer displacement at the point of contact was optimized for minimal cell rupture and maximal monolayer relaxation after tip removal. For MDCK cells, a total displacement of ~50–100 μm was used; for MEKs, displacements of <20 μm were applied (Supplementary Fig. 4 and Supplementary Movie 1). In MEK monolayers, only the cell–cell junction adjacent to the pull was used for subsequent analysis.

Three FRET images were collected for each pull cycle: before tip contact (Pre), while the monolayer was held at maximal displacement (Pull), and 1–5 min after withdrawal of the pipette tip when the monolayer had stopped recoil movement for at least 10 s (Post). Individual puncta were isolated and FRET indices for individual puncta were determined at each time point as described above. To track the motion of puncta in deformed regions, 6–10 corresponding control points were selected manually to generate a projective map with MATLAB’s ‘fitgeotrans’. After this initial warp, a bipartite matching algorithm from MATLAB’s central file exchange (gaimc: Graph Algorithms in Matlab Code) was used to match individual puncta between time points by minimizing the total displacement. Recoil vectors were calculated by matching puncta during the pull to their position post-pull; the length of this vector was defined as the recoil distance *d*_r_. Isolated puncta and puncta that recoiled in a substantially different direction than the average of neighboring puncta were identified as mismatches and excluded.

A second matching was performed between post-puncta to pre-puncta to identify corresponding puncta from before tip contact. To determine the recoil angle *α*_r_, the local cell–cell contact slope was calculated for each punctum by a linear fit through the punctum and all neighboring puncta within 4 μm with MATLAB’s ‘robustfit’; at least three points and a standard error of ≤9° on the resulting slope angle were required. Finally, the acute angle between the cell–cell contact slope (*m*_c_) and the slope of the recoil vector (*m*_r_) was used to determine the angle of the recoil relative to the cell–cell contact, using Eq. (4):

4 $$\alpha _{\mathrm{r}} = {\mathrm{tan}}^{ - 1}\left( {\frac{{m_{\mathrm{r}} - m_{\mathrm{c}}}}{{1 + m_{\mathrm{r}}m_{\mathrm{c}}}}} \right).$$

FRET index differences were determined using a linear mixed-effects model with a grouped effect for puncta obtained from the same image (described in Statistical analysis). The recoil distance- or angle-binned DP-TS data were compared to corresponding, not binned DP-ctrl data.

### Statistical analysis

Statistical tests for change in mean FRET index or FRET efficiency of puncta were performed with R using a linear mixed-effects model with a grouped effect for puncta obtained from the same image. This model accounts for the statistical dependence of puncta from the same image. To this end, grouped error between images is assumed to follow a normal distribution around zero. Including the grouped effect yielded more conservative and robust estimates of statistical confidence relative to tests treating each puncta as independent. The model fretIndex ~ isTensionSensor + (1|imageNumber) was used, where isTensionSensor was set to 1 for the sensor data and 0 for matched truncated control data; imageNumber was a unique index for each image^54^. For drug treatment experiments, a comparison between the solvent control and drug treatment conditions was performed with fretIndex ~ isTensionSensor*isDrugWell + (1|imageNumber) where isDrugWell was set to 0 for the solvent control and 1 for the drug treated conditions. Reported confidence intervals and *p*-values were obtained from the lmer-test package^55^. For statistical evaluation of bulk fitted FLIM data and phospho-MLC quantification, boxplots were generated using MATLAB’s ‘boxplot’ showing the median, the 25th and 75th percentile and whiskers reaching to the last data point within 1.5× interquartile range corresponding to 2.7 standard deviation for normally distributed data. To compare statistical significance, a two-sided Kolmogorov–Smirnov (KS) test with a default significance level of *α* = 0.05 was used because not all data sets satisfied the KS-test for normality. Statistical significances are given by the *p*-value: ***p < 0.001; **p < 0.01; *p < 0.05; n.s. (not significant), *p* ≥ 0.05

### Code availability

Software for TCSPC-FLIM analyses is based on previously published custom-written MATLAB software^17,56^, and was adjusted to this project as described above. Ratiometric FRET analysis was performed using custom-written MATLAB software. Statistical testing was performed using R. Software is available upon request.

## Electronic supplementary material

Supplementary Information

Supplementary Movie

Description of Additional Supplementary Files

Peer Review File

Source Data

Reporting Summary

## Data Availability

Data supporting the findings of this manuscript are available from the corresponding authors upon reasonable request. A Reporting Summary for this Article is available as a Supplementary Information file. The FRET efficiencies, FRET indices, lifetimes, photon counts, puncta numbers, and *p*-values for Figs. 2–5 and Supplementary Figs. 2 and 3 are provided as a Source Data file.
