# Supplementary material for: Mechanical loading of desmosomes depends on the magnitude and orientation of external stress
Source: Nat Commun. 2018 Dec 11;9:5284. doi: 10.1038/s41467-018-07523-0 (PMC6290003; doi:10.1038/s41467-018-07523-0)
Supplement: Supplementary file 1 — Supplementary Information [file 41467_2018_7523_MOESM1_ESM.pdf]

Supplementary Figures for

Mechanical loading of desmosomes depends on magnitude and orientation of external stress

Price, Cost et al.

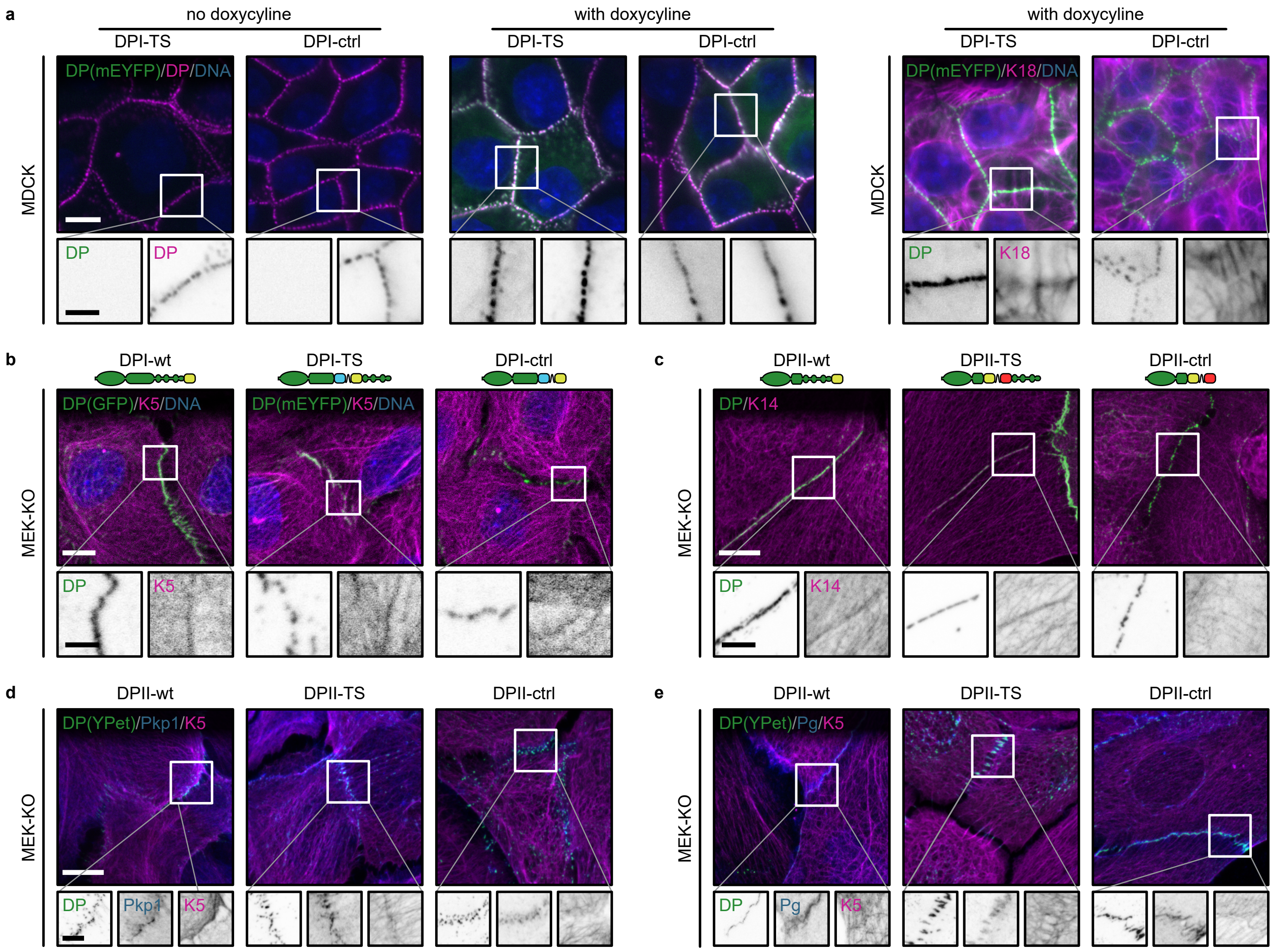

Supplementary Figure 1: Desmoplakin (DP) constructs rescue formation of desmosomes (DSMs). (**a**) DPI construct expression was induced in MDCK cells upon doxycycline addition. DPI constructs co-localized with endogenous DP. Cells expressed keratin (K)-18, as is typical for simple epithelia. (**b, c**) DPI (**b**) and DPII (**c**) constructs localized in DP-deficient keratinocytes (MEK-KO) to cell–cell contacts (images one day after DSM induction). MEKs expressed K5 and K14, as is characteristic of basal keratinocytes; the K5 signal is contrast adjusted. Images are summed projections of nine optical slices covering 3.1 µm. (**d, e**) Puncta containing DPII constructs in rescued MEK-KO one day after DSM induction were also positive for the desmosomal adapter proteins Pkp1 (**d**) and Pg (**e**) indicating formation of *bona fide* DSMs. Images are summed projections of nine optical slices covering 3.1 µm. Scale bars: 10 µm, in zooms: 4 µm.

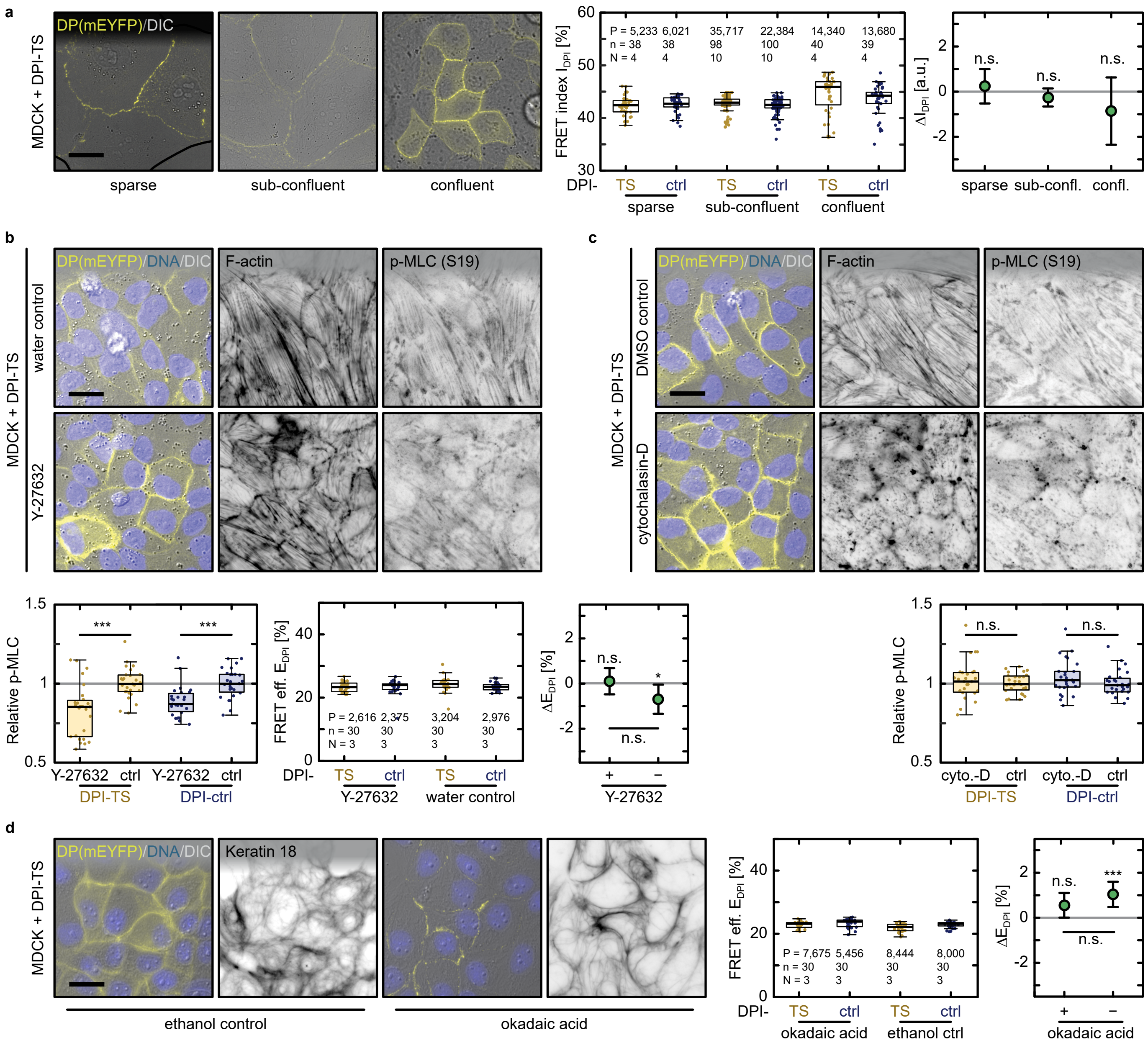

Supplementary Figure 2: DPI tension is negligible under homeostatic conditions and during cytoskeletal disruption. (**a**) MDCK cells were seeded at different densities so that most cells were on an open edge boundary (sparse; boundary indicated by black line), cells formed larger colonies with free edges (sub-confluent), and cells formed monolayers (confluent). As in Fig. 2, the median FRET index value per image is shown as a boxplot, reflecting the broader distribution of individual puncta values used to calculate the mean change in FRET index as $\Delta I = I^{ctrl} - I^{TS}$. (**b**) *Top rows*: MDCK monolayers treated with the ROCK inhibitor Y-27632 showed little apparent change to F-actin architecture compared to matched water control. *Bottom row*: Quantification of the phospho-Myosin Light Chain (p-MLC) immunostaining confirmed a reduction in p-MLC levels upon ROCK inhibition. No changes in tension across DPI in MDCK monolayers were observed. (**c**) *Top rows*: MDCK monolayers treated with the actin destabilizing drug cytochalasin-D showed a disruption of F-actin architecture compared with matched DMSO controls. *Bottom row*: The relative intensity of p-MLC remained unaffected. (**d**) MDCK monolayers treated with okadaic acid showed a disruption of the IF network compared with matched ethanol controls. The mean change between DPI-TS and DPI-ctrl was consistent with no or very little tension in the presence of okadaic acid, and no apparent difference between the ethanol and okadaic acid treated cells. (**a–d**) Boxplots show median, 25th and 75th percentile with whiskers reaching to the last data point within 1.5× interquartile range. ΔI and ΔE plots show mean with 95 % CI; lmer-test: *** $p < 0.001$, * $p < 0.05$, n.s. (not significant) $p \geq 0.05$. Scale bars: 20 µm. (**b–c**) The relative p-MLC intensity was determined by the summed p-MLC intensity divided by the summed DNA intensity (Hoechst 34580). Each experiment was normalized by the average ratio of the matched solvent control experiment. Data from n = 27 images is pooled from N = 3 independent experiments. Boxplots show median, 25th and 75th percentile with whiskers reaching to the last data point within 1.5× interquartile range. Kolmogorov-Smirnov-test: *** $p < 0.001$, n.s. (not significant) $p \geq 0.05$. For detailed p-values, FRET indices, and FRET efficiencies, see Source Data file.

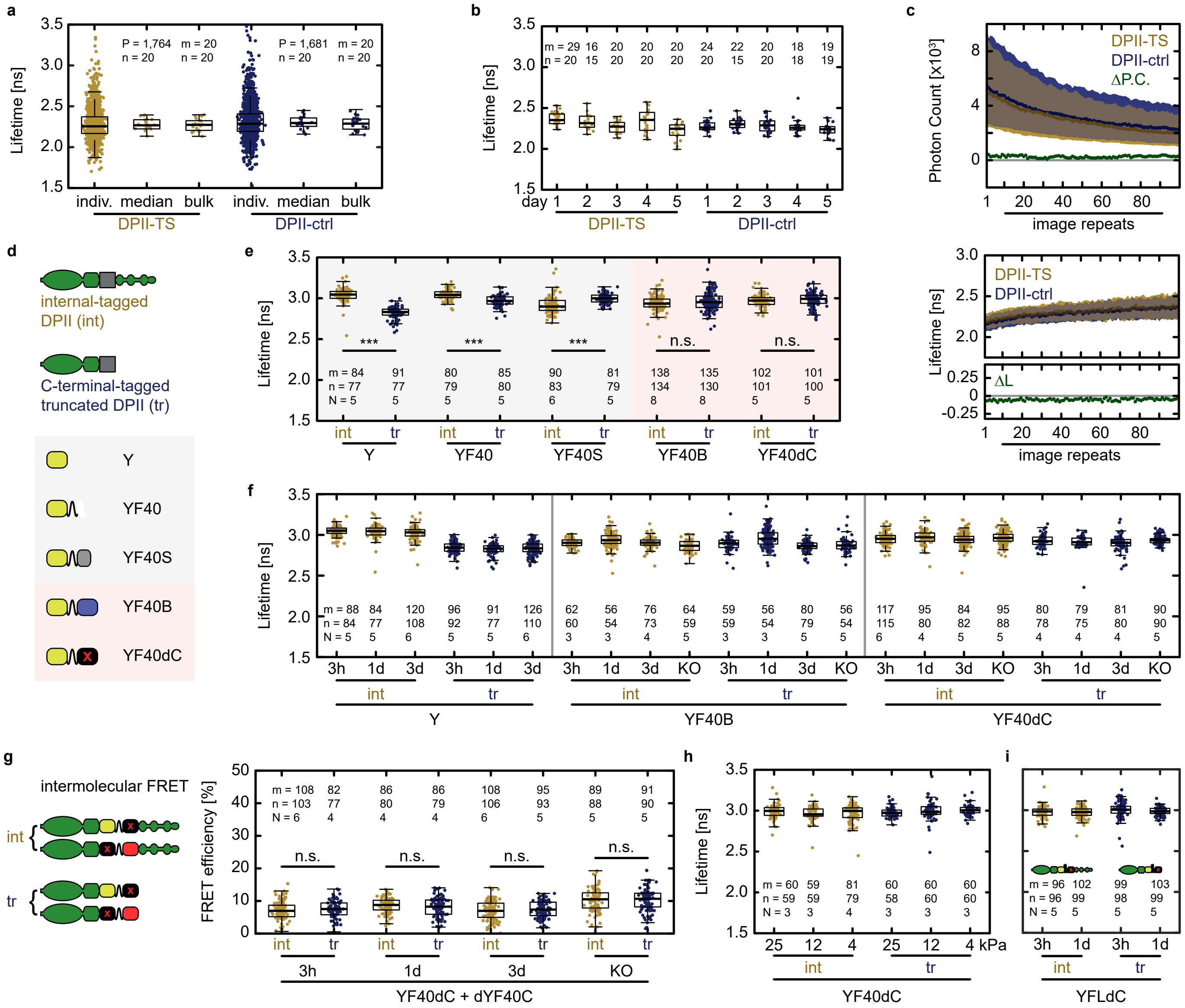

Supplementary Figure 3: FLIM control measurements. (**a**) Fitting of puncta individually, calculating the median per image of these individual fits, or directly fitting in bulk did not change the lifetime for DPII-TS (yellow) or DPII-ctrl (blue). Number of puncta (P), manual masks (m), and images (n) are indicated. (**b**) Lifetime measurements were consistent across experimental days. (**c**) To estimate effects of bleaching, 100 repeat measurements of the same ROI were analyzed. The photon count showed comparable bleaching of YPet for DPII-TS (yellow) and DPII-ctrl (blue); the difference in median photon count (ΔP.C., green) was unchanged; the lifetime increase caused by bleaching did not change the difference in median lifetime (ΔL, green). The median is displayed with interquartile range from m = 391, 404 manual masks from n = 363, 359 images. (**d**) At the integration sites of tension sensor (internal, int; yellow) and control (truncated, tr; blue) constructs, the donor fluorophore was integrated alone (YPet, Y) or with the F40 linker peptide (YF40). In addition, control TSMs were inserted, in which the acceptor was replaced by SNAP-tag (YF40S), TagBFP (YF40B), and Y72L-mutated, non-fluorescent mCherry (YF40dC). Constructs with Y72L-mCherry were used for FRET calculations. (**e**) The donor lifetime of DPII constructs was identical for internal and truncated constructs if the TSM contained non-FRETing fluorophores (i.e. YF40B and YF40dC). (**f**) The donor lifetime did not systematically depend on DSM induction time or the presence of endogenous desmoplakin. (**g**) To determine intermolecular FRET, constructs containing non-fluorescent mCherry (YF40dC) and non-fluorescent YPet (Y67G mutant; dYF40C) were co-expressed. Intermolecular FRET was indistinguishable for internal and truncated constructs. In MEK-KO, FRET efficiencies were elevated. (**h**) Lifetimes for donor only controls were unaffected by the substrate stiffness. (**i**) Lifetimes of constructs containing the FL instead of the F40 linker peptide were undistinguishable and unaffected by the time after DSM induction. (**b, e–i**) Lifetimes and FRET efficiencies were determined via bulk fits. Numbers of manual masks (m), images (n), and independent experiments (N) are indicated in the figure. Boxplots show median, 25th and 75th percentile with whiskers reaching to the last data point within 1.5× interquartile range. Kolmogorov-Smirnov-Test: *** $p < 0.001$, n.s. (not significant) $p \geq 0.05$. For detailed p-values, lifetimes, photon counts, and FRET efficiencies, see Source Data file.

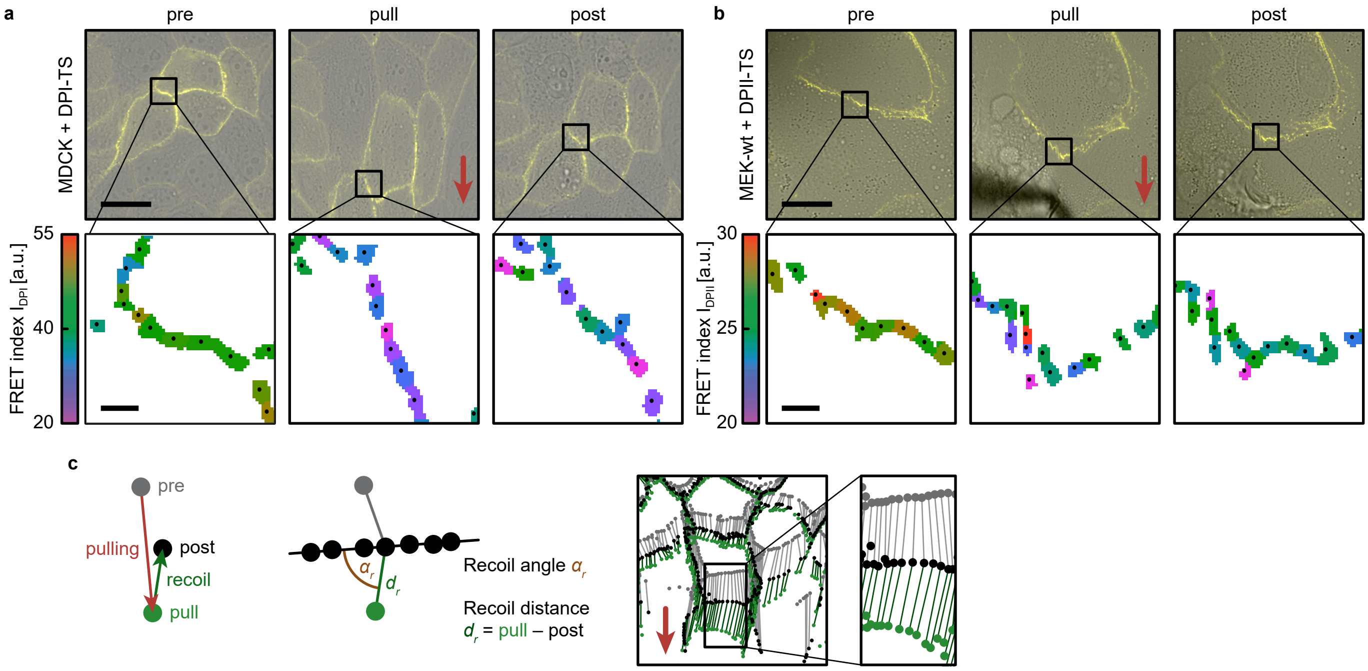

Supplementary Figure 4: Application of mechanical stress to epithelial monolayers. (**a, b**) *Top row*: Images of DPI-TS in confluent MDCK (**a**) and DPII-TS in confluent MEK-wt (**b**) monolayers showing the contrast-adjusted brightfield image superimposed with the fluorescence signal (mEYFP for DPI and YPet for DPII) measured before pulling (pre), at the maximal displacement (pull), and after relaxation of the cell-sheet (post). Scale bar: 20 µm. *Bottom row*: Black dots in the FRET index images mark the position of the centroid used for puncta matching. Scale bar: 2 µm. (**c**) Pre- and pull-puncta were matched to post-puncta. The distance between matched pull- and post-puncta was termed recoil distance d_r and used as a measure for the magnitude of external stress. Similarly, the acute angle between recoil distance and cell–cell contact was termed recoil angle α_r and used as a measure for the orientation of external stress.
