# Supplementary material for: Mechanical loading of desmosomes depends on the magnitude and orientation of external stress
Source: Nat Commun. 2018 Dec 11;9:5284. doi: 10.1038/s41467-018-07523-0 (PMC6290003; doi:10.1038/s41467-018-07523-0)
Supplement: Supplementary file 3 — Description of Additional Supplementary Files [file 41467_2018_7523_MOESM3_ESM.docx]

**Title:**  Supplementary movie 1

**Description:** Example pulling experiments using a glass micropipette to apply mechanical stress to cell monolayers. Four examples are shown in sequence: (1) transmission brightfield and (2) mEYFP fluorescence of DPI-TS expressing MDCK monolayers, and (3) transmission brightfield and (4) mCherry fluorescence of DPII-TS expressing MEK-wt monolayers. Movies are contrast-adjusted. Appearance of a colored dot indicates representative times for FRET image acquisition corresponding to before (grey), during (green), and after the pull (black). Each video represents four minutes in 10x speed. Scale bar: 100 µm.
