# Supplementary material for: Mechanical loading of desmosomes depends on the magnitude and orientation of external stress
Source: Nat Commun. 2018 Dec 11;9:5284. doi: 10.1038/s41467-018-07523-0 (PMC6290003; doi:10.1038/s41467-018-07523-0)
Supplement: Supplementary file 4 — Peer Review File [file 41467_2018_7523_MOESM4_ESM.pdf]

Reviewers' Comments:

Reviewer #1:
Remarks to the Author:

Desmosomes are intercellular adhesive organelles that are especially prominent in tissues that undergo a large amount of mechanical strain. They integrate cells within tissues through interactions with the intermediate filament cytoskeleton, which compared with other types of cytoskeletal systems is quite resistant to mechanical insults. Here, the authors directly assess for the first time the load-bearing properties of desmosomes. By inserting a FRET-based tension sensor module into the intermediate filament anchoring proteins desmoplakins I and II, the authors generated the first sensors to study tension in these adhesive organelles. The authors show that desmoplakin is not under tension during normal homeostasis, unlike other adhesive organelles such as adherens junctions and focal adhesions. It is, however, capable of bearing tension upon external mechanical manipulation. They suggest that the desmosome/intermediate filament system is tuned to withstand external mechanical insults without interfering with normal tissue functions. Overall, the study is well controlled and impactful. The results are likely to provide the basis for many future studies. In addition, the sensors are tools that will be widely useful to investigators interested in the role of desmosomes in mechanics. However, the manuscript requires additional explanation of concepts that are not likely to be easily understood by the wide readership of Nature Communications. In addition, DP-ctl data need to be expressed separately from TS data (mean change in FRET efficiency). These and other comments/suggestions are detailed below.

Comments/suggestions

• The conclusions the authors come to concerning DP experiencing no tension at a basal state are predicated on the assumption that the DP-ctrl constructs cannot experience tension. They present their mean change in FRET efficiency as differences between DP-TS and DP-ctrl constructs. The authors should present their data showing that DP-TS responds to externally applied forces (e.g. Fig 3) separately such that the readers can observe an increase in tension (decrease in FRET) of the DP-TS construct while there were no changes in FRET in the DP-ctrl construct under these same pulling conditions.

• The results presented for mean FRET efficiency exhibit both positive and negative values. The positive values represent tension on DP. However, what negative values could represent is not discussed. Do they represent compression? As multiple figures include negative values (e.g. Fig 2G and Sup. Fig 2B), this needs to be discussed.

• Regarding the assumption that DP-ctrl are incapable of bearing mechanical load because they don't associate with intermediate filaments, the data presented in Fig 1 D and Sup Fig 1 suggest that some intermediate filaments are still anchored to sites of cell-cell adhesion even in MEK-KO cells expressing DP-ctrl, at least at the resolution in the current figures. Either higher resolution fluorescence microscopy or EM (as presented for the full-length DP in Fig 1E) should be included to convince readers that intermediate filaments are indeed uncoupled.

• The control experiments presented in Sup. Fig. 3 should be explained in the text.

• Information about statistical significance should be included in Figure 4.

• The statistical analyses performed need to be better explained. For example, in Sup. Fig. 2 B, there are statistically significant p-values indicated, yet the authors say there are no differences upon addition of drugs. This is especially confusing with the cytochalasin experiments as the value even goes from negative to positive. Please explain.

• The study must include controls showing that the drug treatments, cytochalasin and Y-27632, are effective by showing phalloidin staining/pMLC levels etc. This is essential to appropriately interpret the results as no changes were reported.

• There are subtle differences in the results obtained using DPI vs. DPII. Potential explanations as to why there are differences and/or potential ramifications to biological processes should be discussed, e.g. David Kelsell's work on DPI vs. DPII function in adhesion (JCS 2012). This could also be explained, perhaps, by the different types of keratins expressed in the cell types used, MDCK (K8/18) vs MEKs (K5/14).

Additional detailed comments

• In Figure 1, it could be helpful to the reader if the domains within DP were labeled.
• In Sup. Figure 1A, it would be helpful to label the cells treated with doxycycline, as well.
• In Sup. Figure 4, it would be helpful to add in an arrow indicating the direction of pulling.
• The use of blue to show keratin networks in the micrographs makes it very difficult to interpret. It may be helpful to switch them to magenta and have the other desmosomal proteins pseudocolored to blue. Increasing the size/contrast of the black and white zoomed panels would also be helpful.
• Since there are multiple isoforms of desmogleins and plakophilins, the authors should indicate which they are looking at.
• On line 35- plakoglobins should read plakoglobin.
• All experiments were performed on hard substrates (plastic/glass). Would you predict any differences in the results if softer substrates were used? This could be interesting as substrate rigidity can regulate cell-cell forces.
• On line 202, the authors bring up tension and the taut appearance of intermediate filaments specifically in strained monolayers. However, in the cited study, the monolayers were lifted and had no interactions with a substrate. Under normal cell culture conditions, intermediate filaments (especially keratins) can have a taut appearance, some of which can be seen in this manuscript's figures. Thus, in this case, I would urge caution in using the morphology of the intermediate filaments as an indicator of whether or not the system is under tension or reframe the argument presented.
• Why are the amounts of doxycycline used for induction of DPI-ctrl and DP-TS different?

Reviewer #2:
Remarks to the Author:
Desmosomes are widely known to provide mechanical stability to epithelial tissue, however, little is known about the forces these cell-cell junctions are under. In this work, the authors show that desmosomes are not generally under force and rather exist with a capacity to withstand external forces. This mechanism is different than that of other junctions, which is a fascinating result with broad interest. Understanding the mechanics of desmosomes function is critical, and this is work offers a novel perspective and represents a potentially important contribution. I am enthusiastic, however I do have some concerns that should be addressed.

The DPI and DPII tension sensor constructs should be demonstrated as functional with a cell fragmentation (dispase) assay in the MEK-KO background.

I have concerns with the representation of FRET data. The delta changes measured are very small and need to be better explained. Is the control FRET consistent regardless of treatment or condition? I would like to see the TS and control FRET index or efficiency for each condition plotted for comparison with a statistical comparison. This is important, but obscured by the delta quantification. One area of importance is where the pulling effect is stated to be specific for TS constructs – it is difficult to tell from the histogram data if control changes with pulling or not and I

am not clear if this is shown statistically.

There are times the deltaI/deltaE measurements are negative. This increased FRET efficiency in the TS compared to control needs to be explained in more detail. For example, Figure 2 g MEK-wt 3-5h is significantly lower than the other data points. Does this mean the tension is increased in the other 3 conditions compared to 3-5 h? This is not the only example, supplemental Figure 2 also shows this for several conditions. If this is due to subtle changes in architecture as posited by the authors then can the FRET measurement be trusted to report DP tension? It is generally assumed all FRET is between the donor-acceptor on one DP. How would FRET between different DP molecules impact the measurements? Given desmosome architecture, is FRET between different DPs possible? How would this impact interpretation of FRET data?

How do the FRET values compare to off puncta and background in both the lifetime and sensitized emission experiments?

Can the pN tensions experienced by DP in junctions under stress be calculated? How do these relate to tension in other adhesive structures?

Minor points

Page 2 line 57 This note is best understood only after reading the manuscript, there is no mention of model systems before this point.

Page 3 line 87 It is not clear what is meant by “network architecture”

Reviewer #3:
Remarks to the Author:
The manuscript by Price and colleagues addresses a long-standing question of whether desmosomes are mechanically sensitive cell-cell junctions. Desmosomes are long known to protect epithelia against mechanical stress through their connections with the intermediate filament network in cells. Using a well-characterized method of FRET-based molecular tension sensors, Price and colleagues present evidence in support of this idea. The authors used extensive quantitative assays to characterize the desmoplakin based force sensors to demonstrate a role for the desmosomes-IF system in force sensing. They conclude that desmoplakins (I and II) experience mechanical force during acute extrinsic deformative stress but not due to cell-intrinsic forces due to actomyosin contractility. The experimental design is excellent with appropriate controls and the data is convincing. Overall, this is a technically-expert study which introduces reagents that will undoubtedly be useful for the field. However, it is not clear that, in its current form, it represents the conceptual advance that would appeal to the general audience of Nature Communications.

The major concerns are:
1. The authors base the mechanical loading of Desmoplakins (I and II) on their ability to couple with intermediate filament system. However, the molecular details of the differences in observed FRET-based TSM readout under basal conditions versus deformative stress are unclear. Importantly, whether the observed difference in mechanical loading under these conditions arise primarily due to differences in desmosome-IF coupling under these conditions. The use of DP mutants to strengthen its binding with IF may provide useful insights in this direction.

2. The assembly and organization of IF system in cells is dependent on actin and microtubule network. It is interesting to address whether these cytoskeletal networks contribute to the mechanical loading and/or coupling of DSM-IF system, especially under deformative stress.

3. The IF network is extensively remodelled and reinforced during external stress and is critical for mechanical resilience of epithelia. It is interesting and important to address whether stress induced IF remodelling is essential for mechanical loading of desmosomes. Mutations interfering with the IF re-organization (rod domain or PTMs at head/tail domains) may provide valuable insights into this.

4. It is interesting to observe that the mechanical load on DSM positively correlates with the magnitude of deformative stress. However, the molecular reason for this is unclear. Is it because of increased coupling of DSM with IF network or is it because of increased molecular load experienced by DSM under a constant DSM-IF coupling, as the authors claim?

Minor point:

1. A technical concern; The authors consistently use and compare the difference between FRET index of DPI-ctrl and DPI-TS as a measure of mechanical load on DP. However, It is crucial to gauge the range of DPI-TS independent of its comparison with DPI-ctrl. In this regard, it is important to know the FRET index difference with DP-TS upon the acute collapse of IF network (e.g., Okadaic acid) and long-term KD or KO of the intermediate filament network.

Response to Reviewer #1

Desmosomes are intercellular adhesive organelles that are especially prominent in tissues that undergo a large amount of mechanical strain. They integrate cells within tissues through interactions with the intermediate filament cytoskeleton, which compared with other types of cytoskeletal systems is quite resistant to mechanical insults. Here, the authors directly assess for the first time the load-bearing properties of desmosomes. By inserting a FRET-based tension sensor module into the intermediate filament anchoring proteins desmoplakins I and II, the authors generated the first sensors to study tension in these adhesive organelles. The authors show that desmoplakin is not under tension during normal homeostasis, unlike other adhesive organelles such as adherens junctions and focal adhesions. It is, however, capable of bearing tension upon external mechanical manipulation. They suggest that the desmosome/intermediate filament system is tuned to withstand external mechanical insults without interfering with normal tissue functions. Overall, the study is well controlled and impactful. The results are likely to provide the basis for many future studies. In addition, the sensors are tools that will be widely useful to investigators interested in the role of desmosomes in mechanics. However, the manuscript requires additional explanation of concepts that are not likely to be easily understood by the wide readership of Nature Communications. In addition, DP-ctl data need to be expressed separately from TS data (mean change in FRET efficiency). These and other comments/suggestions are detailed below.

Response: We wish thank the reviewer for the careful review of our manuscript and the supportive and constructive remarks. In response to the comments, we performed experiments and included new data sets, and we rearranged figures and adjusted the main text. The manuscript is markedly improved as a result, and we hope that the reviewer can now fully support the publication of our study.

• The conclusions the authors come to concerning DP experiencing no tension at a basal state are predicated on the assumption that the DP-ctrl constructs cannot experience tension. They present their mean change in FRET efficiency as differences between DP-TS and DP-ctrl constructs. The authors should present their data showing that DP-TS responds to externally applied forces (e.g. Fig 3) separately such that the readers can observe an increase in tension (decrease in FRET) of the DP-TS construct while there were no changes in FRET in the DP-ctrl construct under these same pulling conditions.

Response: In addition to plotting data as a FRET differential between DP-TS and DP-ctrl, we now also show the individual FRET indices in Fig. 4 and Fig. 5. The data show that mechanical pulling causes a specific decrease in DP-TS FRET indices whereas DP-ctrl values are largely unaffected.

• The results presented for mean FRET efficiency exhibit both positive and negative values. The positive values represent tension on DP. However, what negative values could represent is not discussed. Do they represent compression? As multiple figures include negative values (e.g. Fig 2G and Sup. Fig 2B), this needs to be discussed.

Response: We show that the negative values do not originate from changes in intermolecular FRET (Supplementary Fig. 3g), but whether isolated negative delta values (e.g. Fig. 2d) are caused by compression or biased fluorophore orientation is unclear. We now mention in the main text that negative FRET values could originate from altered fluorophore orientation or compression of the F40 peptide, as indeed has been proposed in the past (Paszek et al., *Nature*, 2014; Rothenberg et al., *Cell Mol. Bioeng.*, 2015; both studies are now cited in the main text).

To ensure that measurements at 3-5 h after DSM induction are valid and to corroborate our interpretation that DP does not bear tension under homeostatic conditions, we generated a new set of DP tension sensors using our recently developed FL-TS (Ringer et al., *Nature Methods*, 2017). This sensor module is based on a different mechanosensitive peptide but also responds to low forces of 3-5 pN. Analyzing cells expressing the FL-TS-based constructs indicates that DP is not exposed to tension at 3-5 h. These data are now included in Fig. 2f. We also repeated FRET measurements of drug treated cells represented in the former Supplementary. Fig. 2B using FLIM instead of ratiometric imaging (Fig. 2b and Supplementary. Fig. 2b). This helped control for possible confounding effects (e.g. background variation associated with drug pipetting and the incubation times required). These measurements confirmed a lack of effect specific to drug addition compared to solvent controls.

• Regarding the assumption that DP-ctrl are incapable of bearing mechanical load because they don't associate with intermediate filaments, the data presented in Fig 1 D and Sup Fig 1 suggest that some intermediate filaments are still anchored to sites of cell-cell adhesion even in MEK-KO cells expressing DP-ctrl, at least at the resolution in the current figures. Either higher resolution fluorescence microscopy or EM (as presented for the full-length DP in Fig 1E) should be included to convince readers that intermediate filaments are indeed uncoupled.

Response: As suggested by the reviewer, we performed EM analysis of MEK-KO cells expressing the DP-ctrl construct. A representative image of these experiments is now included in Fig. 1e. The data support the notion that the interaction of DSMs, and DP specifically, with IFs is impaired in DP-ctrl expressing cells.

• The control experiments presented in Sup. Fig. 3 should be explained in the text.

Response: We extended the main text to explain the data presented in Supplementary Fig. 3 in more detail.

• Information about statistical significance should be included in Figure 4.

Response: Detailed statistical information for all experiments is now included in new Excel files, denoted as Supplementary Table 1-6. They are associated to individual figures. In addition, we added a shaded box to the absolute value curves indicating the 95% confidence interval of the control data.

• The statistical analyses performed need to be better explained. For example, in Sup. Fig. 2 B, there are statistically significant p-values indicated, yet the authors say there are no differences upon addition of drugs. This is especially confusing with the cytochalasin experiments as the value even goes from negative to positive. Please explain.

Response: We agree that the data in Supplementary Fig. 2 and their presentation was somewhat confusing; in a nutshell we felt at the time that the FRET differences, while statistically significant were unlikely to represent biologically meaningful changes in tension. To address this point, we repeated the measurements after drug treatment using FLIM, which is both more accurate and provides absolute FRET efficiencies rather than a FRET index. The new experiments (now shown in Fig. 2 and Supplementary Fig. 2) confirm that the addition of cytochalasin-D and Y-27632 does not affect tension across DP under the

tested conditions. The section on statistical analysis now describes in more detail how significance tests specific for the effect of drug addition were performed.

• The study must include controls showing that the drug treatments, cytochalasin and Y-27632, are effective by showing phalloidin staining/pMLC levels etc. This is essential to appropriately interpret the results as no changes were reported.

Response: We thank the reviewer for drawing our attention to this. To verify the effectiveness of the drugs employed, we performed immunostainings of solvent control and drug-treated cells using a Ser-19 phospho-MLC (p-MLC) antibody and co-labelled the cells with phalloidin. The intensity of p-MLC staining was used as a measure of ROCK activity, and the phalloidin signal to determine the effectiveness of the cytochalasin-D treatment. The quantifications show that Y-27632 lead to the expected decrease in ROCK activity, whereas cytochalasin-D treatment disrupts actin stress fiber integrity. The data are shown in Supplementary Fig. 2b, c.

• There are subtle differences in the results obtained using DPI vs. DPII. Potential explanations as to why there are differences and/or potential ramifications to biological processes should be discussed, e.g. David Kelsell's work on DPI vs. DPII function in adhesion (JCS 2012). This could also be explained, perhaps, by the different types of keratins expressed in the cell types used, MDCK (K8/18) vs MEKs (K5/14).

Response: This is a very interesting point. Indeed, our data would be consistent with some level of DP-isoform specificity and we extended the discussion accordingly; also the work from David Kelsell is now mentioned. In addition, to directly investigate this point, we performed DPI pulling experiments in MEKs. In contrast to DPII-measurements, effects on DPI loading were moderate even at higher recoil distances. These data further support the hypothesis of a DP-isoform specific effect in keratinocytes and they are now shown in Fig. 4c. We thank the reviewer for bringing this point into focus—the role of different DP isoforms in mediating tension transmission specifically in skin will be an interesting topic for future investigations in our, and hopefully other, laboratories.

• In Figure 1, it could be helpful to the reader if the domains within DP were labeled.

Response: The individual domains are now labelled.

• In Sup. Figure 1A, it would be helpful to label the cells treated with doxycycline, as well.

Response: We labelled images of the doxycycline treated cells accordingly.

• In Sup. Figure 4, it would be helpful to add in an arrow indicating the direction of pulling.

Response: We inserted a red arrow to indicate the direction of pulling.

• The use of blue to show keratin networks in the micrographs makes it very difficult to interpret. It may be helpful to switch them to magenta and have the other desmosomal proteins pseudocolored to blue. Increasing the size/contrast of the black and white zoomed panels would also be helpful.

Response: We adjusted the colors and increased the size of the zoomed panels allowing an easier interpretation of the data.

• Since there are multiple isoforms of desmogleins and plakophilins, the authors should indicate which they are looking at.

Response: We now specifically state in the main text that Desmoglein-1/2 and plakophilin-1 were visualized.

• On line 35- plakoglobins should read plakoglobin.

Response: This has been corrected.

• All experiments were performed on hard substrates (plastic/glass). Would you predict any differences in the results if softer substrates were used? This could be interesting as substrate rigidity can regulate cell-cell forces.

Response: We thank the reviewer for raising this interesting point. We performed experiments on substrates covering surface rigidities of 2, 4, 12, and 25 kPa using FLIM. The new data (now shown in Fig. 3a-c) show that DP is not exposed to mechanical tension on substrates ≥4 kPa. However, we observed DP tension on very soft substrates (2 kPa). We believe that these results are specific as DP-TS expressing cells displayed reduced FRET efficiencies when adherent to the center of the 2 kPa dish but not when adhering to the surrounding stiff rim in the same dish. These experiments suggest that DP can become exposed to mechanical tension on very soft substrates. As it is not entirely clear whether the 2 kPa condition reflects a physiological relevant stiffness (basement membranes and epidermal tissue were reported to be more rigid), we are careful with the interpretation of this data set. The effect may be relevant in upper layers of the epidermis, which are not in contact with the basement membrane and have been reporter to be softer.

• On line 202, the authors bring up tension and the taut appearance of intermediate filaments specifically in strained monolayers. However, in the cited study, the monolayers were lifted and had no interactions with a substrate. Under normal cell culture conditions, intermediate filaments (especially keratins) can have a taut appearance, some of which can be seen in this manuscript's figures. Thus, in this case, I would urge caution in using the morphology of the intermediate filaments as an indicator of whether or not the system is under tension or reframe the argument presented.

Response: The reviewer is right; the taut appearance of IFs in itself is probably not a good indicator of mechanical tension. We therefore deleted this part of the sentence.

• Why are the amounts of doxycycline used for induction of DPI-ctrl and DP-TS different?

Response: Different amounts of doxycycline were used to obtain comparable expression levels of DP-TS and DP-ctrl. This is now mentioned in the methods section. We found that expression levels tended to be

higher in DP-ctrl expressing cells (see Reviewer #1 Fig.1), thus doxycyline concentrations were reduced in this case.

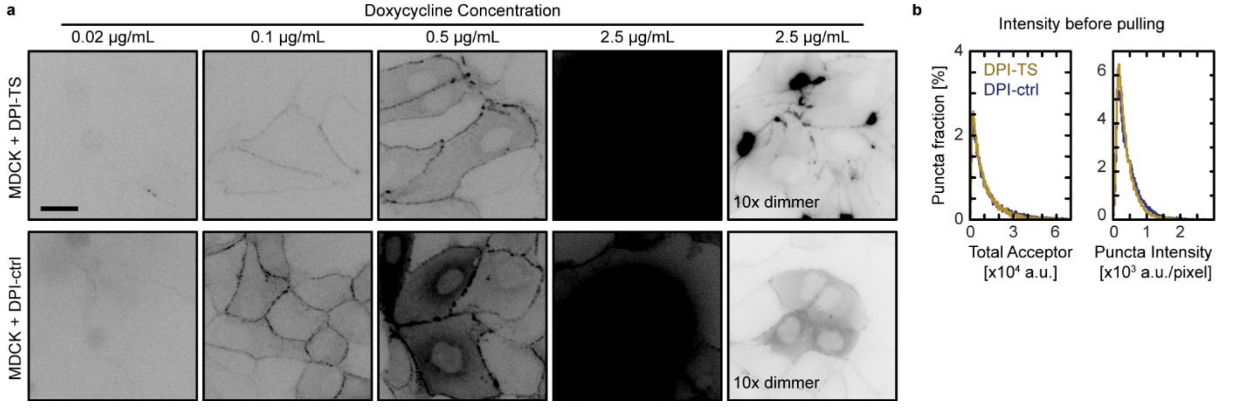

Reviewer #1 Fig.1: Different doxycycline concentrations were compared*. (**a**) DPI-ctrl required less doxycycline to induce a comparable expression level as for DPI-TS. Scale bar: 20 µm. (**b**) The acceptor intensities of all MDCK cell sheets imaged before pulling are highly comparable for DPI-TS and DPI-ctrl demonstrating that the chosen conditions (0.5 µg/ml for DPI-TS and 0.1 µg/ml for DPI-ctrl) indeed resulted in similar expression levels.*

Response to Reviewer #2

Desmosomes are widely known to provide mechanical stability to epithelial tissue, however, little is known about the forces these cell-cell junctions are under. In this work, the authors show that desmosomes are not generally under force and rather exist with a capacity to withstand external forces. This mechanism is different than that of other junctions, which is a fascinating result with broad interest. Understanding the mechanics of desmosomes function is critical, and this is work offers a novel perspective and represents a potentially important contribution. I am enthusiastic, however I do have some concerns that should be addressed.

Response: We wish to thank the reviewer for carefully reviewing our manuscript and providing thoughtful and constructive comments. We have addressed the raised issues, performed additional experiments and made the requested changes in the main text. In addition, we included additional data sets requested by other reviewers, we extended the main text, methods section, and we rearranged figures. As a result, the manuscript is substantially improved and we hope that the reviewer can support the publication of our study.

The DPI and DPII tension sensor constructs should be demonstrated as functional with a cell fragmentation (dispase) assay in the MEK-KO background.

Response: We agree with the reviewer that it would be ideal to document DP functionality with the dispase assay. Therefore, we tried for several years to adapt this assay to confirm DP-TS functionality in MEK-KO cells. Unfortunately, it was not possible to obtain sufficiently high transfection efficiencies in this cell system in order to perform the assay reliably. The problem is that DP tension sensor plasmids are very large (about 14 kDa) and therefore difficult to express, especially in keratinocytes. We could obtain maximal transfection efficiencies of about 30%, which was not sufficient to perform the assays. Even positive control constructs (wt-desmoplakin) were not able to rescue MEK-KO cells. We note that similar observations have been published. For instance, studies with keratin-deficient cells revealed that in a mixture of keratin-wt and keratin-deficient cells at least 60% wild type keratinocytes are required for cell-cell adhesion that can be detected with the dispase assay (Bär et al., *J Invest Dermatol,* 2014). We therefore could not include this experiment into the manuscript.

As an alternative, we present extensive data showing that our DP constructs rescue DSM formation and ultrastructure in MEK-KO cells (Fig. 1). Based on these data we are confident that the DP tension sensors rescue, at a minimum, the function of DP in linking DSMs to the IF cytoskeleton.

I have concerns with the representation of FRET data. The delta changes measured are very small and need to be better explained. Is the control FRET consistent regardless of treatment or condition? I would like to see the TS and control FRET index or efficiency for each condition plotted for comparison with a statistical comparison. This is important, but obscured by the delta quantification. One area of importance is where the pulling effect is stated to be specific for TS constructs – it is difficult to tell from the histogram data if control changes with pulling or not and I am not clear if this is shown statistically.

Response: We apologize that the data were not presented in this format to begin with—we struggled with how best to present the data in full while still making it interpretable to non-specialists. We now show

FRET indices of DP-TS and DP-ctrl before, during and after pulling (Fig. 4 and Fig. 5). The new presentation demonstrates that the control construct is largely insensitive to experimental treatments while the DP-TS constructs responds in a specific fashion.

There are times the deltaI/deltaE measurements are negative. This increased FRET efficiency in the TS compared to control needs to be explained in more detail. For example, Figure 2 g MEK-wt 3-5h is significantly lower than the other data points. Does this mean the tension is increased in the other 3 conditions compared to 3-5 h? This is not the only example, supplemental Figure 2 also shows this for several conditions. If this is due to subtle changes in architecture as posited by the authors then can the FRET measurement be trusted to report DP tension? It is generally assumed all FRET is between the donor-acceptor on one DP. How would FRET between different DP molecules impact the measurements? Given desmosome architecture, is FRET between different DPs possible? How would this impact interpretation of FRET data?

Response: Please also see our response to Reviewer 1 on this point. In brief, increased FRET in Fig. 2g (now Fig. 2d) could potentially represent either compression of the F40 linker peptide or a subtle environmental effect on the relative orientation of the fluorophores, and these possibilities are mentioned in the text. However, to further explore this point, we generated a set of DP tension sensors using our recently developed FL-TS (Ringer et al., *Nature Methods*, 2017). This sensor module is based on a different mechanosensitive peptide but also responds to very low forces of about 3-5 pN. Analyzing cells expressing FL-TS-based constructs confirmed no tension at any time point and we did not observe negative FRET values at the 3-5 h time point. The new data are now included in Fig. 2f. Thus, the most parsimonious interpretation is that DP is not under tension at the 3-5 h timepoint.

Earlier experiments with drugs aimed at disrupting the actin cytoskeleton were performed using ratiometric FRET. We repeated these measurements using FLIM, a more accurate technique that also provides absolute FRET values. In addition, we performed immunostainings to demonstrate the efficiency of the drug treatments and included another drug, okadaic acid. Together, the experiments demonstrate that the addition of cytochalasin-D or Y-27632 or okadaic acid does not affect tension across DP under the tested conditions. As suggested by the reviewer, we now also show that the negative FRET difference are not caused by differences in intermolecular FRET (Supplementary Fig. 3g).

How do the FRET values compare to off puncta and background in both the lifetime and sensitized emission experiments?

Response: We determined the off puncta and background FRET values for FLIM measurements (shown here in Reviewer #2 Fig.1). As expected, off puncta signals show significantly reduced photon count rates and an increased spread in the data. The overall FRET values are not dramatically different from junctional puncta values because both are dominated by signals from unloaded DP sensors. Fitting of cytosolic signals further increased data spread while unspecific signal from extracellular areas leads to completely aberrant values.

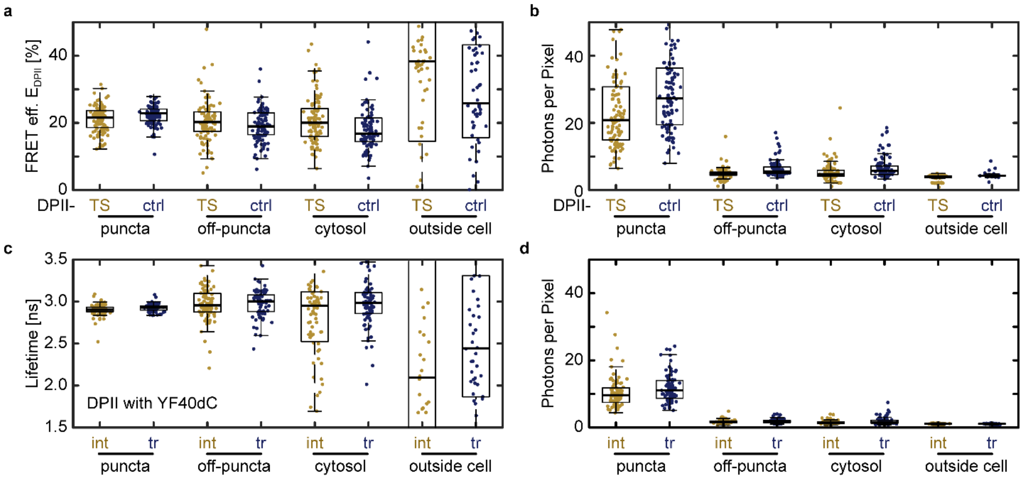

***Reviewer #2 Fig.1: Example analysis of non-puncta background for F40-based DPII constructs in MEK-wt one day after DSM induction**. "Puncta" is a bulk fit per image of the data included in the paper, "off-puncta" contains all signal inside the manual mask that is excluded during thresholding, "cytosol" is the unthresholded signal in a separate cytosolic mask, and "outside cell" is the unthresholded signal in a separate mask drawn on neighboring untransfected cells. FRET efficiencies of DPII-TS (yellow) and DPII-ctrl (blue) (**a**) as well as lifetimes of donor only controls (**c**) are shown with the corresponding photon count per pixel (**b, d**).*

For the ratiometric measurements, the background subtraction leads to the appearance of negative intensity values for some of the pixels in the diffuse/dim parts of the image, which complicate the data analysis. This makes it difficult to directly address to the review's interest in FRET values for cytoplasmic DP in the context of this experiment.

As an alternative, we examined puncta from the cytoplasm of each region of interest (see Reviewer #2 Fig. 2). These may represent misannotated junctional DSMs, DSM components that have been endocytosed, or other structures. The resulting data are noisy, and difficult to definitively interpret. However, it appears that a subset of these puncta may show reduced FRET values during pulling. Given the available evidence, the simplest explanation would be a misannotation of some DSMs (tilted cell-cell junctions sometimes make it difficult to unambiguously identify all of the DSMs associated with a junction). However, given the noisiness of these data and the attendant ambiguities we would prefer to not include this analysis in the manuscript.

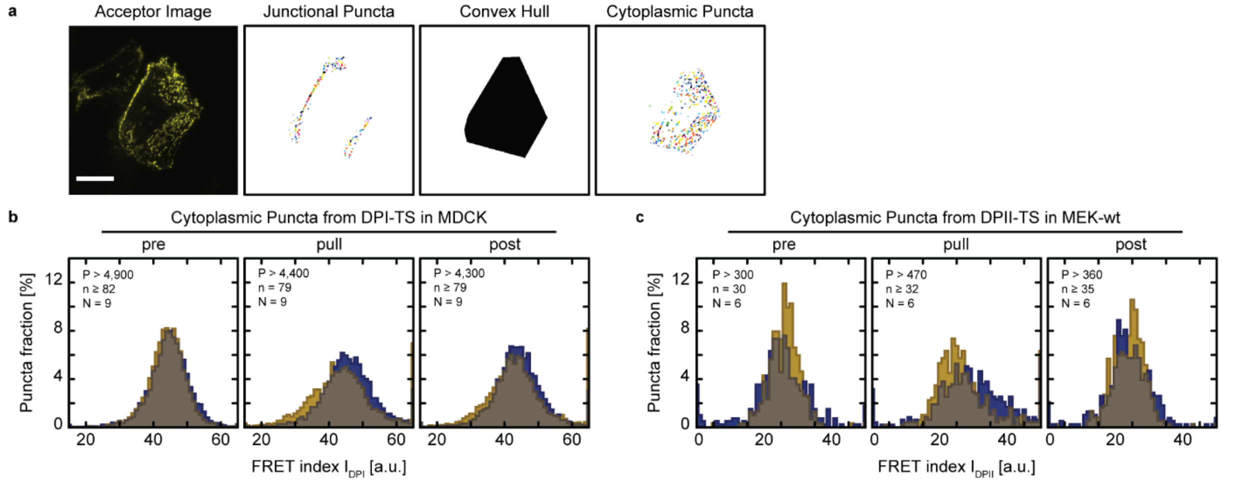

Reviewer #2 Fig.2: ***Example analysis of cytoplasmic puncta in ratiometric FRET data****. (**a**) From the acceptor image, a junctional puncta mask was used to extract the relevant signal analyzed in Fig. 3-5. To determine contributions from the cytoplasm of the cell, puncta extraction was performed on the convex hull of the junctional puncta (excluding the previously identified junctional puncta). Scale bar: 20 µm. (**b**) FRET index from cytoplasmic puncta in the DPI-TS pulling experiment with MDCK monolayers shows a similar trend as the junctional puncta, but larger proportion of extreme values (>65). (**c**) Similarly, FRET index from cytoplasmic puncta in the DPII-TS pulling experiment with MEK-wt shows a similar trend as the junctional puncta but a larger proportion of extreme values (>50). Puncta numbers (P), number of images (n), and experiments (N) are indicated.*

Can the pN tensions experienced by DP in junctions under stress be calculated? How do these relate to tension in other adhesive structures?

Response: Ensemble measurements only allow for an estimate of the average FRET per molecule but not the calculation of force per molecule, as discussed previously (Freikamp, Cost, Grashoff, *Trends in Cell Biol*, 2016). For a general sense of scale, a force of 2 pN evenly distributed across all molecules would yield a FRET index change of approximately ΔI_{DPI}~7.5 or ΔI_{DPII}~5 for the respective F40-based tension sensors. These values are similar to the changes in FRET index observed for puncta undergoing large deformations (Figs. 4 and 5). Our anticipation is thus that desmosomes bear loads similar to, for example, E-cadherin (Borghi *et al., PNAS,* 2012), but unlike E-cadherin do so only when external load is applied. However, a comprehensive study employing additional complementary, calibrated sensors will be required before a quantitative comparison to other systems can be well supported. For this reason, we have been conservative in ascribing pN tension values to our FRET measurements.

Relatedly, please note the special case in which we can straightforwardly relate FRET to tension, namely that zero difference in FRET between TS and ctrl constructs ($\Delta I = 0$) corresponds to zero tension. This lack of tension at steady-state appears to be a major distinguishing feature of the desmosome relative to other adhesion structures measured to date.

Page 2 line 57 This note is best understood only after reading the manuscript, there is no mention of model systems before this point.

Response: We now write “genetic mouse models”.

Page 3 line 87 It is not clear what is meant by “network architecture

Response: We changed this to “desmosomal architecture”.

Response to Reviewer #3

The manuscript by Price and colleagues addresses a long-standing question of whether desmosomes are mechanically sensitive cell-cell junctions. Desmosomes are long known to protect epithelia against mechanical stress through their connections with the intermediate filament network in cells. Using a well-characterized method of FRET-based molecular tension sensors, Price and colleagues present evidence in support of this idea. The authors used extensive quantitative assays to characterize the desmoplakin based force sensors to demonstrate a role for the desmosomes-IF system in force sensing. They conclude that desmoplakins (I and II) experience mechanical force during acute extrinsic deformative stress but not due to cell-intrinsic forces due to actomyosin contractility. The experimental design is excellent with appropriate controls and the data is convincing. Overall, this is a technically-expert study which introduces reagents that will undoubtedly be useful for the field.

However, it is not clear that, in its current form, it represents the conceptual advance that would appeal to the general audience of Nature Communications.

Response: We thank the reviewer for carefully reviewing our manuscript, for the encouraging comments, and for providing very interesting suggestions. We agree that additional mechanistic insight would further strengthen the manuscript. Therefore, we performed additional control experiments, analyzed a newly generated DP mutant construct, generated a new DP force sensor (DPII-TS-FL), and analyzed the effect of substrate stiffness on force transmission through DP (see responses to reviewer 1 and 2). These extensive new data add to the biological insight derived from our study, further strengthening a study that was already judged to be “impactful” and an “important contribution” by reviewers 1 and 2. Given the expanded scope of our study, we are hopeful that the reviewer can now concur with these assessments.

1. The authors base the mechanical loading of Desmoplakins (I and II) on their ability to couple with intermediate filament system. However, the molecular details of the differences in observed FRET-based TSM readout under basal conditions versus deformative stress are unclear. Importantly, whether the observed difference in mechanical loading under these conditions arise primarily due to differences in desmosome-IF coupling under these conditions. The use of DP mutants to strengthen its binding with IF may provide useful insights in this direction.

Response: To investigate a potential isoform-specific role of desmoplakin, we performed DPI measurements in MEKs. These experiments revealed that, indeed, DPI is less sensitive to externally applied forces compared to DPII in keratinocytes. We therefore generated and analyzed a DPI-TS construct harboring the S2849G mutation, which is expected to increase DP-IF coupling. Intriguingly, mechanical tension across the DPI-mutant was not increased under steady-state conditions, and the DPI-mutant response was very similar to that of wt DPI-TS in pulling experiments. These data are now included in Fig. 4c, d) and indicate that an increase in DSM-IF coupling alone is not sufficient to load DPI.

2. The assembly and organization of IF system in cells is dependent on actin and microtubule network. It is interesting to address whether these cytoskeletal networks contribute to the mechanical loading and/or coupling of DSM-IF system, especially under deformative stress.

Response: This is a very interesting suggestion. However, addressing this point in a detailed manner would involve the use of at least two independent drugs for both actin and MT networks. Together with the

requests of the other two reviewers, it would have been impossible to perform the combined cytoskeletal disruption/pulling experiments in a reasonable amount of time. However, we mention in the discussion that this interesting issue could be addressed by the biosensors developed in this study.

3. The IF network is extensively remodelled and reinforced during external stress and is critical for mechanical resilience of epithelia. It is interesting and important to address whether stress induced IF remodelling is essential for mechanical loading of desmosomes. Mutations interfering with the IF re-organization (rod domain or PTMs at head/tail domains) may provide valuable insights into this.

Response: While certainly very interesting, we believe that this question is best addressed in the context of a separate, full-length study. The required experiments necessitate the generation of cell lines containing both mutant keratins and the DP force sensor, and which would then need to be characterized using all of the control measurements and cytoskeletal disruptor experiments described in the present work.

Please note, however, that the new data included in the revision provide considerable additional insight into the closely related question of when and under what circumstances DSMs bear load: *i*) We now report that culturing keratinocytes on soft substrates leads to a basal level of tension on DPII. *ii*) We show that a mutation altering the affinity of DPI for IFs does *not* lead to increased tension, suggesting that, at least in the circumstances studied, the kinetics of the DP-keratin interaction are not critical in setting load levels. *iii*) Finally, we show that disruption of the IF cytoskeleton due to treatment with okadaic acid does not lead to decreased (or increased) DPI FRET. These new data thus extend our former understanding of when DSMs transmit tension between neighboring cells.

Relatedly, in preliminary work we have identified a keratin head domain mutation previously used with keratin-18 (R90C) as a potential means for disrupting keratin assembly in MDCK cell monolayers (Ku et al., *J Cell Biol*, 1995). We derived double-stable cell lines expressing DPI-TS (downstream of a constitutive EF1α promoter) and Keratin18-PSmOrange or Keratin-18(R90C)-PSmOrange in an inducible manner (downstream of an inducible TRE promoter). Our preliminary results with these cell lines revealed significant heterogeneity in phenotype, where overexpression of either normal or mutant keratin sometimes induced the collapse of the IF network and sometimes did not (see Reviewer #3 Fig.1). These observations give early suggestions of possible challenges in the (doubtless interesting) experiments that the reviewer proposes.

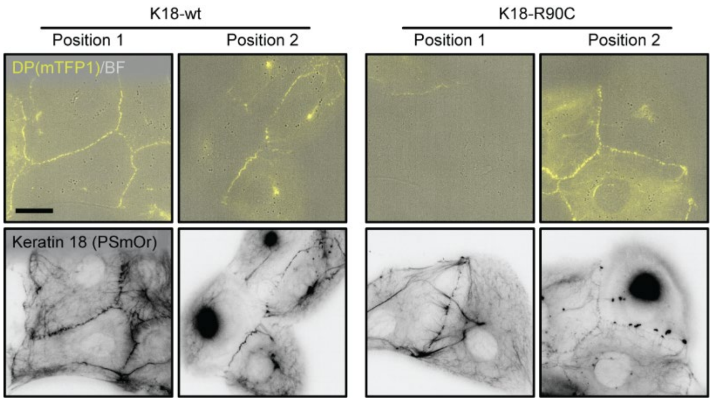

Reviewer #3 Fig.1: Modulating keratin assembly. *Example from double-stable cell lines with constitutive DPI-TS expression and inducible keratin-18 (K18-wt, left) or mutated keratin-18 (K18-R90C, right) expression. Two images from the same well for each line are shown in order to demonstrate the heterogeneous response in both cell lines. Also note heterogeneous expression of the DPI-TS sensor in these cell lines. Scale bar: 20 µm.*

4. It is interesting to observe that the mechanical load on DSM positively correlates with the magnitude of deformative stress. However, the molecular reason for this is unclear. Is it because of increased coupling of DSM with IF network or is it because of increased molecular load experienced by DSM under a constant DSM-IF coupling, as the authors claim?

Response: Our new experiments reveal an interesting isoform specificity. In keratinocytes, the correlation of deformation and DP tension is especially pronounced for DPII, whereas DPI seems less sensitive. We inserted the S2849G mutation into DPI to test whether an increased DSM-IF coupling would lead to stronger correlation of deformation and DPI tension in MEKs, but our experiments suggest that this is not the case.

We note, however, that ensemble measurements with our live-cell tension sensors cannot easily distinguish between increased coupling and increased molecular load. The newly developed FL-based DP tension sensor is in principle capable of this distinction at the specific force threshold of ~4 pN. However, such tests need to be complemented by experiments in which different tension sensor modules are compared with each other, as we have shown earlier for talin-1 (Ringer et al., *Nature Methods*, 2017). We believe that such experiments would require a better-controlled and more parallelizable mechanism for applying strain. While we do not currently have the expertise to establish such a device, we are confident that our sensors could help address these fascinating mechanistic questions, and thus look forward to our sensors being more widely available.

1. A technical concern; The authors consistently use and compare the difference between FRET index of DPI-ctrl and DPI-TS as a measure of mechanical load on DP. However, It is crucial to gauge the range of DPI-TS independent of its comparison with DPI-ctrl. In this regard, it is important to know the FRET index difference with DP-TS upon the acute collapse of IF network (e.g., Okadaic acid) and long-term KD or KO of the intermediate filament network.

Response: As suggested by the reviewer, we performed the okadaic experiment, which is now included in Supplementary Fig. 2d. The experiments show that okadaic acid treatment does not significantly change FRET values compared to control conditions, confirming the interpretation that DP does not experience tension under homeostatic cell culture conditions.

Reviewers' Comments:

Reviewer #1:
Remarks to the Author:
The authors have done an excellent and thorough job of addressing the majority of the reviewers comments. The revised manuscript includes an extensive amount of new data, including a new version of the DP tension sensor, the IF binding-enhanced S2849G DP mutant, and an experiment testing the effects of substrate rigidity on DP tension, among others. In addition, the authors now present DP-control data separately from DP TS data, which greatly aides in data interpretation. Overall, the manuscript is now suitable for publication in Nature Communications. However, authors should consider addressing one issue prior to acceptance. During the revision process, Daniel Conway's group published a study in Cells that showed that the desmosomal cadherin desmoglein 2 (Dsg2) is under tension in both cardiac cells and epithelial cells (including MDCK cells that are used in the present study). The authors of the present study do include this Dsg2 study as a new reference in the revised manuscript, but do not discuss how it relates to their own findings that DP is not under tension unless exposed to external forces. A more thorough discussion about how the Dsg2 findings fit into the present study as well as potential explanations about the seemingly disparate outcomes should be included.

An addition minor note: Sup. Fig 1 d and e- the color coding for PKP1/PG and K5 appear to be switched.

Reviewer #2:
None

Reviewer #3:
Remarks to the Author:
I appreciate the efforts of the authors to address the concerns and suggestions of the reviewers. The new experiments in fig 5 provide additional insights into the isoform-specific role of DP in bearing mechanical load. However, the manuscript still lacks an explanation for one of the fundamental observation by the authors and this has to be addressed before the manuscript is accepted for publication.

1. The key point that was not addressed was how DP is mechanically loaded only upon external stress or when the cells are grown on soft substrates (<2 PKa). I appreciate the efforts of the authors for performing the experiments in Fig 4 - to test the strength of DP-IF coupling for mechanical loading of DPI-TS. However, the absence of difference in FRET in this DPI mutant is intriguing (Fig 4d) as the authors largely base the differences in the FRET efficiency, observed on DPI-TS and DPII-TS upon external stress, to the coupling of DPI and II to keratin intermediate filaments (KIFs). Instead, this potentially may reflect the altered mechanical properties of KIF upon externally applied stretch (Sivaramakrishnan et al., PNAS 2008), apart from the DP-IF coupling. An important question, therefore, is whether the DP-TS (I and/or II) could sense and report the changes in mechanical load upon external stress especially when the mechanical properties of KIFs are affected. I appreciate that the authors attempted to use keratin 18 mutant (R90C) to induce a dominant negative effect, however, did not pursue the experiments with this mutant. Mutations blocking the reorganization capabilities of KIFs (eg., K18S33A) are known to exhibit normal network architecture at steady state (Sivaramakrishnan et al., Mol Biol Cell 2009, Ku et al., JBC 2002), but have a huge impact on the properties and mechanical functions of KIFs during stress response.

2. Are the FRET values of DP-TS are different if the intermediate filament network is acutely perturbed during stress or in cells on the soft substrate?

3. It will be useful to indicate the desmosomes in the zoomed images of EM in fig 1e.

4. It will be useful to number individual plots in Fig 2.

5. Figure numbers should be cited at Line 138 and 142.

Response to Reviewer # 1

The authors have done an excellent and thorough job of addressing the majority of the reviewers comments. The revised manuscript includes an extensive amount of new data, including a new version of the DP tension sensor, the IF binding-enhanced S2849G DP mutant, and an experiment testing the effects of substrate rigidity on DP tension, among others. In addition, the authors now present DP-control data separately from DP TS data, which greatly aides in data interpretation.

Response: We thank the reviewer for the constructive and supportive remarks throughout this review process. We feel that the reviewer's comments made a significant contribution to improving the manuscript.

Overall, the manuscript is now suitable for publication in Nature Communications. However, authors should consider addressing one issue prior to acceptance. During the revision process, Daniel Conway's group published a study in Cells that showed that the desmosomal cadherin desmoglein 2 (Dsg2) is under tension in both cardiac cells and epithelial cells (including MDCK cells that are used in the present study). The authors of the present study do include this Dsg2 study as a new reference in the revised manuscript, but do not discuss how it relates to their own findings that DP is not under tension unless exposed to external forces. A more thorough discussion about how the Dsg2 findings fit into the present study as well as potential explanations about the seemingly disparate outcomes should be included.

Response: We now discuss the recent Conway paper in a new paragraph of the discussion. We offer one potential biological explanation for the apparently contrasting results of our studies, namely force transduction across alternative linkages.

An addition minor note: Sup. Fig 1 d and e- the color coding for PKP1/PG and K5 appear to be switched.

Response: The text color now matches the image color coding.

Response to Reviewer #3

I appreciate the efforts of the authors to address the concerns and suggestions of the reviewers. The new experiments in fig 5 provide additional insights into the isoform-specific role of DP in bearing mechanical load. However, the manuscript still lacks an explanation for one of the fundamental observation by the authors and this has to be addressed before the manuscript is accepted for publication.

1. The key point that was not addressed was how DP is mechanically loaded only upon external stress or when the cells are grown on soft substrates (<2 PKa). I appreciate the efforts of the authors for performing the experiments in Fig 4 - to test the strength of DP-IF coupling for mechanical loading of DPI-TS. However, the absence of difference in FRET in this DPI mutant is intriguing (Fig 4d) as the authors largely base the differences in the FRET efficiency, observed on DPI-TS and DPII-TS upon external stress, to the coupling of DPI and II to keratin intermediate filaments (KIFs). Instead, this potentially may reflect the altered mechanical properties of KIF upon externally applied stretch (Sivaramakrishnan et al., PNAS 2008), apart from the DP-IF coupling. An important question, therefore, is whether the DP-TS (I and/or II) could sense and report the changes in mechanical load upon external stress especially when the mechanical properties of KIFs are affected. I appreciate that the authors attempted to use keratin 18 mutant (R90C) to induce a dominant negative effect, however, did not pursue the experiments with this mutant. Mutations blocking the reorganization capabilities of KIFs (eg., K18S33A) are known to exhibit normal network architecture at steady state (Sivaramakrishnan et al., Mol Biol Cell 2009, Ku et al., JBC 2002), but have a huge impact on the properties and mechanical functions of KIFs during stress response.

Response: We thank the reviewer for the detailed comments and interesting suggestions. We agree that open questions remain regarding the molecular mechanisms underlying force transmission at desmosomes. However, we believe that a comprehensive investigation of this topic is beyond the scope of the current study. Please note that we developed two novel desmoplakin sensors, and developed experimentally challenging protocols to demonstrate that desmoplakin is mechanically loaded upon external stress. Understanding how cells remodel KIFs and desmosomes in response to mechanical stress requires experiments that include, and go considerably beyond, the experiments suggested by the reviewer. To facilitate progress in the field as a whole, it seems best publishing this study and stimulating also other research groups to address many of these questions.

We now highlight in the discussion the importance of future studies that employ mutations in keratin that affect KIF reorganization to emphasize this point.

2. Are the FRET values of DP-TS are different if the intermediate filament network is acutely perturbed during stress or in cells on the soft substrate?

Response: While this is an interesting issue, the proposed experiment is of uncertain feasibility given the relative crudeness of the available reagents that can acutely influence IF architecture. More fundamentally, we believe that a separate, comprehensive study is required to properly investigate the issue of how IF network remodeling affects force transmission (please see our response to point 1).

3. It will be useful to indicate the desmosomes in the zoomed images of EM in fig 1e.

Response: Desmosomes and intermediate filaments are now indicated in the zoomed images.

4. It will be useful to number individual plots in Fig 2.

Response: Because the delta and boxplots function as two complementary representations of the same dataset, we would prefer to keep the numbering of subsections of Figure 2 unchanged in order to allow for a concise description in the figure legend.

5. Figure numbers should be cited at Line 138 and 142.

Response: Figure numbers are now cited as requested.
